# Selected Nutraceuticals in Metabolic Syndrome: Molecular Mechanisms and Clinical Implications

**DOI:** 10.3390/biomedicines14030646

**Published:** 2026-03-12

**Authors:** Josè Starvaggi, Carla Di Chio, Fabiola De Luca, Santo Previti, Maria Zappalà, Roberta Ettari

**Affiliations:** Department of Chemical, Biological, Pharmaceutical, and Environmental Sciences, University of Messina, Viale Ferdinando Stagno d’Alcontres 31, 98166 Messina, Italy; jose.starvaggi@studenti.unime.it (J.S.); carla.dichio@unime.it (C.D.C.); fabiola.deluca@unime.it (F.D.L.); spreviti@unime.it (S.P.); mzappala@unime.it (M.Z.)

**Keywords:** nutraceuticals, chemoprevention, metabolic syndrome, cardiovascular diseases

## Abstract

In recent years, there has been a growing scientific and clinical interest in nutraceuticals, bioactive compounds derived from natural sources such as plants, fruits and cereals. These substances have gained prominence due to their diverse pharmacological properties, particularly their anti-inflammatory, antioxidant and antitumor activities. In addition, scientific evidence supports their beneficial role in the prevention and management of cardiovascular diseases, which represent the principal focus of the present review. This review provides a comprehensive and detailed analysis of selected nutraceuticals related to the metabolic syndrome, a multifactorial pathological condition characterized by a cluster of metabolic disturbances that collectively increase the risk of developing cardiovascular disease and type 2 diabetes. The metabolic syndrome is typically defined by the presence of abdominal obesity, insulin resistance, hypertension and dyslipidemia, which includes elevated plasma triglyceride levels and decreased concentrations of high-density lipoprotein (HDL) cholesterol. Given the global importance and prevalence of metabolic syndrome, identifying new strategies to treat these disorders, such as the use of nutraceuticals, has become a central focus of biomedical research.

## 1. Introduction

The concept of metabolic syndrome was first introduced in 1988 as “Syndrome X” to describe the frequent clustering of insulin resistance with metabolic abnormalities that increase the risk of type 2 diabetes mellitus and cardiovascular disease [1,2]. The term was later refined to “metabolic syndrome” to avoid confusion with cardiac Syndrome X and to better reflect the underlying metabolic dysregulation [3]. Over the years, several international organizations including the World Health Organization (WHO), the European Group for the Study of Insulin Resistance (EGIR), the National Cholesterol Education Program Adult Treatment Panel III (NCEP ATP III), the American Association of Clinical Endocrinology (AACE), and the International Diabetes Federation (IDF) have proposed diagnostic criteria that differ primarily in the emphasis placed on insulin resistance versus central obesity [4,5,6,7,8,9,10]. Earlier definitions (WHO, EGIR, AACE) focused mainly on insulin resistance as a mandatory component, whereas later frameworks prioritized anthropometric and metabolic parameters that are more easily applicable in clinical practice.

Currently, the most widely accepted and clinically relevant diagnostic framework is the Harmonized Definition, jointly developed by major international organizations [11]. This definition identifies metabolic syndrome based on the presence of at least three of the following components: increased waist circumference (population specific), hypertriglyceridemia and/or reduced HDL cholesterol, elevated blood pressure, and impaired fasting glucose [12,13]. Waist circumference is used as a practical surrogate marker of visceral adiposity and insulin resistance, as direct measurement of insulin levels is not feasible in routine clinical screening [14].

Accumulating evidence indicates that fat distribution rather than total adiposity is the principal determinant of metabolic risk. Individuals with predominant abdominal (visceral) fat accumulation exhibit a higher likelihood of insulin resistance and cardiometabolic complications compared to those with peripheral fat distribution [15,16,17]. For this reason, waist circumference is often interpreted alongside BMI or waist-to-height ratio has become a cornerstone of metabolic syndrome diagnosis [14,18]. Moreover, visceral obesity is closely associated with ectopic fat deposition, including non-alcoholic fatty liver disease, further reinforcing its clinical relevance.

## 2. Methods

We performed a narrative literature review to identify publications reporting on the epidemiology, pathophysiology, and nutritional and nutraceutical-based management of metabolic syndrome. A comprehensive search of PubMed/MEDLINE, Web of Science, Scopus was conducted from database. Search terms included combinations of the following keywords: “metabolic syndrome,” “insulin resistance,” “obesity,” “dyslipidemia,” “hypertension,” “nutraceuticals,” “functional foods,” “dietary supplements,” “macronutrients,” “bioactive compounds,” “polyphenols,” “fatty acids,” “plant sterols,” “vitamins,” “minerals,” and “metabolites.”

We included original research articles (randomized controlled trials, observational studies, and experimental studies), clinical trials, and systematic and narrative reviews that addressed the role of dietary components, nutraceuticals, or food-derived metabolites in the development, prevention, or management of metabolic syndrome. Articles not published in English and studies lacking sufficient methodological or outcome-related information were excluded.

Given the narrative nature of the review, the heterogeneity of study designs, and the broad scope of the topic, no formal quality assessment or meta-analysis was performed.

## 3. Metabolic Syndrome in Different Populations

### 3.1. Metabolic Syndrome in Adults

Metabolic syndrome is a highly prevalent clinical condition in adults, characterized by the clustering of interconnected metabolic abnormalities that significantly increase the risk of type 2 diabetes mellitus (T2DM), cardiovascular disease (CVD), and all-cause mortality. In adults, metabolic syndrome is commonly defined by the coexistence of central obesity, insulin resistance, dyslipidemia (elevated triglycerides and reduced high-density lipoprotein cholesterol), hypertension, and impaired glucose metabolism. The prevalence of metabolic syndrome increases markedly with age and is strongly influenced by lifestyle factors such as physical inactivity, unhealthy dietary patterns, and excess caloric intake. Pathophysiologically, visceral adiposity plays a central role by promoting chronic low-grade inflammation, oxidative stress, and adipokine dysregulation, which collectively contribute to insulin resistance and endothelial dysfunction [19].

### 3.2. Metabolic Syndrome in Older Adults

Metabolic syndrome is diffused among older adults and represents a major contributor to age-related morbidity and mortality. Aging is associated with profound metabolic changes, including increased visceral adiposity, sarcopenia, reduced insulin sensitivity, chronic low-grade inflammation, and endothelial dysfunction, all of which predispose elderly individuals to metabolic syndrome. Epidemiological data indicate that the prevalence of metabolic syndrome rises steadily with age, exceeding 40–50% in individuals over 60 years, depending on diagnostic criteria and population studied. In older adults, it is strongly associated with an increased risk of cardiovascular disease, type 2 diabetes mellitus, cognitive decline, frailty, and reduced functional capacity [20].

### 3.3. Metabolic Syndrome in Children and Adolescents

As with adults and older adults, there is no consensus regarding the definition of metabolic syndrome in children and adolescents [21]. In 2007, the IDF published a report [22] that included three age groups: for children aged 6 to 9 the metabolic syndrome is defined as the presence of abdominal obesity, with waist circumference ≥ 90th percentile; between ages of 10 to 15 the definition includes abdominal obesity (waist circumference ≥ 90th percentile, or if lower, adult cut-off values), plus two or more of the following criteria, hypertriglyceridemia and/or low HDL cholesterol (triglycerides ≥ 150 mg/dL; HDL cholesterol < 40 mg/dL), hypertension (systolic/diastolic blood pressure ≥ 130/85 mmHg) and type 2 diabetes mellitus or impaired fasting glucose (≥100 mg/dL). Over the age of 16 the definition follows adult criteria, i.e., the presence of abdominal obesity (waist circumference ≥ 94 cm for European males and ≥80 cm for European females—ethnicity-specific cut-off values apply), plus two or more of the following hypertriglyceridemia and/or low HDL cholesterol (triglycerides ≥ 150 mg/dL; HDL < 40 mg/dL in males and <50 mg/dL in females), hypertension (≥130/85 mmHg) and type 2 diabetes mellitus or impaired fasting glucose (≥100 mg/dL). However, pediatricians are primarily concerned that all current definitions of metabolic syndrome in children are derived from adult definitions. The predictive value and clinical utility of applying adult criteria to younger populations have not been fully established. Indeed, while it is hypothesized that metabolic syndrome in childhood is related to metabolic syndrome in adulthood, this relationship has not yet been conclusively demonstrated. Studies linking childhood metabolic syndrome to adult cardiovascular disease are still quite limited. Another limitation is that these definitions do not consider the influence of growth and puberty for instance, the “normal” insulin resistance seen during puberty [23,24], changes in fat and lean body mass, growth, and the secretion of sex steroids. It has been shown that insulin resistance in obese children progressively increases through the Tanner stages and is consistently higher than in their normal-weight peers throughout puberty [25]. Additional factors not included in the current definitions are gestational age, birth weight, breastfeeding, parental obesity, and family medical history [26]. Thus, diagnosing metabolic syndrome in childhood is complex [27]. Several studies on adolescent cohorts have shown that metabolic syndrome is not a stable diagnosis during adolescence. For example, factor analysis and the examination of developmental transitions [28,29] indicated that the diagnosis can vary based on maturity level. Further research on children and adolescents aged from 8 to 16 years has shown that pubertal obese children are more likely to have partial or full metabolic syndrome than their prepubertal peers [30]. Data from the Bogalusa Heart Study and cardiovascular risk studies in Finnish youth [31] indicate that while children and adolescents with metabolic syndrome are at increased risk for adult metabolic syndrome, atherosclerosis, and type 2 diabetes, BMI alone is an equally accurate indicator for identifying those at risk for adult metabolic syndrome and later atherosclerosis. These limitations suggest that prevention and treatment of metabolic syndrome in children and adolescents should focus more on established risk factors rather than on diagnosis, which remains inconsistent due to growth and pubertal influences [31]. While nutraceuticals such as polyphenols, plant proteins, omega-3 fatty acids, and vitamins have been investigated for their potential benefits in managing metabolic syndrome in children and adolescents, it is critical to evaluate both safety and long-term efficacy in this vulnerable population. Pediatric metabolism, growth trajectories, and endocrine regulation differ markedly from adults, raising concerns that chronic supplementation could inadvertently affect linear growth, pubertal progression, and hormonal balance. Moreover, although short-term studies often report improvements in BMI, fasting glucose, and lipid profiles, data on sustained metabolic benefits, cardiovascular risk reduction, and long-term outcomes remain limited [31].

Such an approach would more effectively reduce BMI and cardiovascular risk in the long term.

## 4. Nutraceuticals for the Prevention of Metabolic Syndrome

A wide range of nutraceuticals has been investigated for the prevention of metabolic syndrome. Among these, macronutrients, polyphenols, berberine, fermented red rice, α-lipoic acid, benfotiamine, fucoxanthin, policosanols, and plant sterols have emerged as the most extensively studied, demonstrating substantial experimental and clinical evidence supporting their beneficial effects on metabolic parameters. The selection of compounds discussed in this review reflects both their prevalence in the literature and their relevance to key pathophysiological mechanisms underlying metabolic syndrome.

### 4.1. Macronutrients

The most well-known macronutrients for the treatment of metabolic syndrome are represented by plant proteins, amino acids and fatty acids.

#### 4.1.1. Plant Proteins

##### Dietary Role and Epidemiological Evidence

Protein-rich diets are increasingly used as a strategy for weight management and the prevention of type 2 diabetes mellitus [32,33]. However, the source and quality of protein are critical. High intake of red and processed meat has been linked to increased risk of type 2 diabetes and cardiovascular disease [34], whereas consumption of plant-based proteins is generally associated with reduced risk of type 2 diabetes, dyslipidemia, and metabolic syndrome [35].

Epidemiological studies have shown that increased dietary intake of vegetable proteins, including soy, lupin, peas, and wheat proteins, is inversely correlated with the incidence of metabolic syndrome components, such as hyperglycemia, elevated triglycerides, and abdominal obesity [35,36]. Among these, lupin and soy proteins have been the most extensively studied in humans, demonstrating potentially positive effects on metabolic parameters and cardiovascular risk [36].

##### Lupin Proteins: Composition, Mechanisms, and Effects

Lupin is a legume obtained from species such as *Lupinus albus*, *L. luteus*, *L. mutabilis*, and *L. angustifolius*. Lupin seeds are rich in proteins (up to 40%) and have minimal anti-nutritional factors [37]. Lupin proteins are primarily composed of 7S and 11S globulins and conglutins (α, β, γ, δ), which have been extensively studied for their metabolic effects.

Lipid Metabolism

Lupin proteins (Table 1) reduce plasma LDL cholesterol and improve the LDL:HDL ratio by upregulating hepatic LDL receptor expression, mainly through the SREBP-2 (sterol regulatory element-binding protein 2) pathway [38,39,40]. They also reduce plasma levels of PCSK9, a key negative regulator of LDL receptors, by 12.7% in human studies [41,42,43]. Animal models further confirm hypolipidemic and anti-atherosclerotic effects, supporting their cardiovascular benefits [44].

Glucose Metabolism

Lupin proteins, particularly γ-conglutin, reduce postprandial glycemia in both animal and human studies [44]. Mechanistically, lupin-derived peptides stimulate IRS-1 phosphorylation, activating the PI3K/Akt signaling pathway, which promotes GLUT4 translocation to the plasma membrane in skeletal muscle and adipocytes, enhancing glucose uptake and insulin sensitivity. Hydrolyzed lupin peptides (Lup1) have also been shown to inhibit DPP-IV, a molecular target associated with type 2 diabetes mellitus [45,46,47].

Anti-inflammatory and Lipid Oxidation Effects

Lupin proteins inhibit NF-κB signaling in adipocytes and hepatocytes, lowering pro-inflammatory cytokines such as TNF-α and IL-6. They also activate AMP-activated protein kinase (AMPK), enhancing fatty acid oxidation and reducing hepatic lipogenesis [38,40].

##### Soy Proteins: Composition, Mechanisms, and Effects

Soy (*Glycine max*) is a high-quality plant protein source, often considered the prototype of plant-based proteins [48]. It also contains isoflavones, which exhibit estrogen-like and anti-atherosclerotic effects [49].

Lipid and Cholesterol Regulation

Soy proteins (Table 1) activate PPARα in hepatocytes, upregulating genes involved in fatty acid β-oxidation and decreasing triglyceride synthesis. They enhance LDL receptor expression via SREBP-2, improving LDL clearance and reducing plasma cholesterol [50]. In humans, soy protein supplementation reduces triglycerides by 15–20% in individuals with mild hypertriglyceridemia, regardless of isoflavone content [51].

Glucose Homeostasis

Soy derived bioactives stimulate IRS-1 phosphorylation and the PI3K/Akt pathway, facilitating GLUT4 translocation in muscle and adipose tissue, improving glucose uptake and insulin sensitivity. Comparative studies with lupin proteins in patients with type 2 diabetes indicate similar glycemic control effects [52].

Body Weight and Metabolic Syndrome Components

Long term observational and interventional studies, such as the EPIC-PANACE study, demonstrate that replacing animal protein with soy protein reduces body weight gain, total cholesterol, LDL cholesterol, and other metabolic syndrome components in men over several years [53,54].

Additional Mechanisms

Isoflavones in soy mimic estrogen activity, enhancing endothelial function and vascular health. Soy proteins also promote satiety, reduce cholesterol absorption, and inhibit angiotensin-converting enzyme (ACE), contributing to blood pressure regulation.

##### Integrated Mechanisms of Plant Proteins in Metabolic Syndrome

Both lupin and soy proteins contribute to the management of metabolic syndrome through multiple mechanisms: Improvement of lipid profiles: Upregulation of LDL receptors, reduced PCSK9, enhanced fatty acid oxidation. Enhanced glucose homeostasis: Activation of IRS-1/PI3K/Akt signaling, increased GLUT4 translocation, DPP-IV inhibition. Anti-inflammatory and antioxidant effects: Inhibition of NF-κB signaling, reduction in pro-inflammatory cytokines. Cardiovascular benefits: Isoflavone-mediated endothelial protection, ACE inhibition, anti-atherosclerotic effects. Weight management: Increased satiety and reduction in energy intake.

These effects collectively mitigate the core pathophysiological features of metabolic syndrome, including insulin resistance, dyslipidemia, hypertension, and chronic inflammation.

#### 4.1.2. Amino Acids

Amino acids play critical roles not only as building blocks of proteins but also as metabolic regulators, influencing glucose homeostasis, lipid metabolism, and energy balance. Imbalances in circulating amino acids have been linked to components of metabolic syndrome, including insulin resistance, dyslipidemia, and central obesity [55,56]. Among these, branched-chain amino acids (BCAAs; leucine, isoleucine, valine) and non-essential amino acids such as alanine have been most extensively studied for their metabolic effects.

Elevated plasma BCAAs have been associated with insulin resistance and type 2 diabetes risk, whereas alanine plays a role in gluconeogenesis and energy metabolism, which may influence fasting glucose and liver fat accumulation Therefore, understanding the mechanistic and clinical effects of these amino acids is crucial for evaluating their potential role in metabolic syndrome prevention or management.

##### Alanine

**Figure 1:**
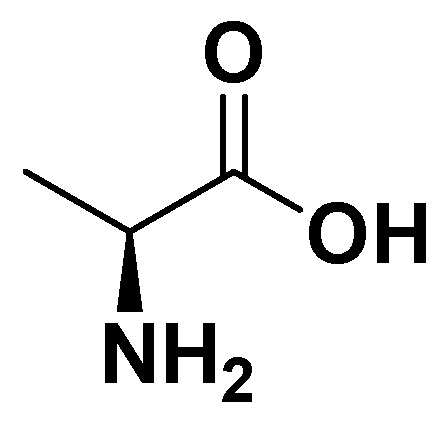
Alanine.

Alanine (Figure 1) is a non-essential amino acid that participates in the glucose–alanine cycle, transporting nitrogen from muscle to the liver and providing substrates for gluconeogenesis. Elevated alanine levels have been associated with hepatic insulin resistance and non-alcoholic fatty liver disease (NAFLD), both of which are key components of metabolic syndrome. Alanine can modulate hepatic glucose production by supplying pyruvate for gluconeogenesis. Clinical studies have suggested that dietary interventions reducing excessive alanine flux, such as protein-balanced diets, may improve fasting glucose and insulin sensitivity, although evidence from RCTs remains limited (Table 1).

##### Leucine

**Figure 2:**
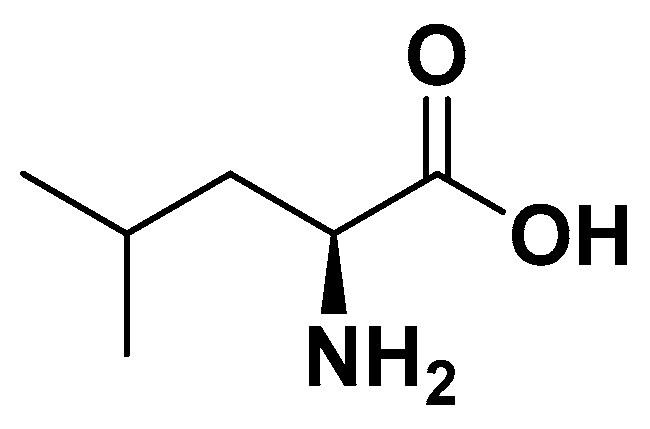
Leucine.

Leucine (Figure 2) activates the mTOR (mechanistic target of rapamycin) signaling pathway, promoting protein synthesis and muscle glucose uptake. Leucine also stimulates insulin secretion and modulates AMPK activity, enhancing fatty acid oxidation and improving lipid profiles (Table 1). 

##### Valine

**Figure 3:**
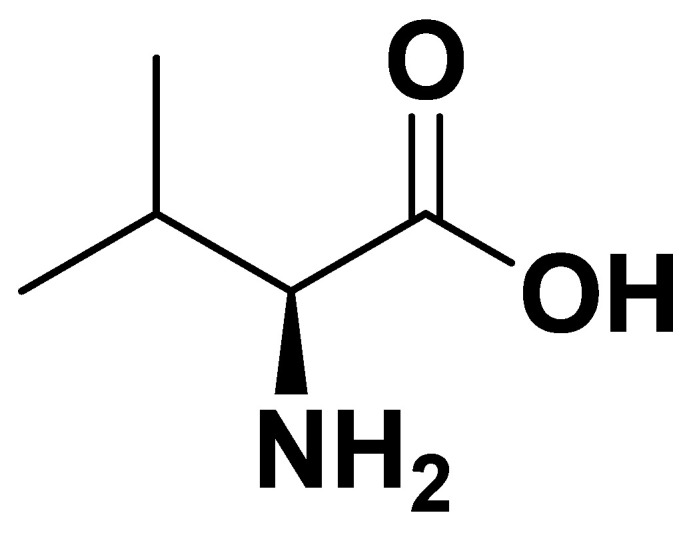
Valine.

Valine (Figure 3), like other BCAAs, contributes to energy metabolism through branched-chain α-ketoacid dehydrogenase (BCKDH)-mediated catabolism, generating substrates for the TCA cycle. Elevated plasma valine levels are often observed in insulin-resistant and obese individuals, suggesting its potential as a biomarker of metabolic dysfunction (Table 1). 

#### 4.1.3. Fatty Acids

Dietary fats play a central role in the modulation of metabolic syndrome components, including lipid profiles, glycemic control, and inflammation. The impact of fats depends largely on their chemical structure, chain length, and degree of saturation. Studies indicate that both the quality and type of dietary fats, rather than total fat quantity, are critical determinants of metabolic outcomes [57,58,59].

##### Saturated Fatty Acids

**Figure 4:**
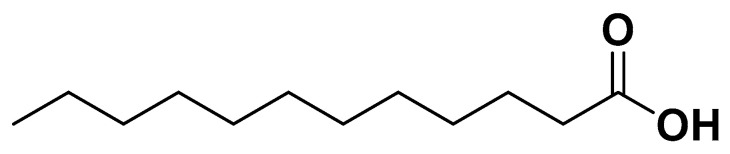
Lauric acid.

Saturated fatty acids (SFAs) have traditionally been associated with increased cardiovascular risk, particularly long-chain SFAs such as palmitic acid [60]. However, medium-chain saturated fatty acids (MCFAs), such as lauric acid, caprylic acid, and capric acid, exhibit different metabolic properties. Coconut oil, which is rich in MCFAs (caprylic acid 8%, capric acid 7%, lauric acid 49%), has demonstrated antithrombotic, anti-inflammatory, and lipid-modulating effects [61,62,63]. Lauric acid (Figure 4) specifically improves cardiovascular risk markers and body composition compared to other SFAs [64]. Virgin coconut oil is preferred over refined oil due to its higher phenolic content, providing antioxidant protection and mitigating oxidative stress [62].

MCFAs enhance fatty acid oxidation and modulate lipid metabolism, reducing hepatic lipogenesis. Additionally, dietary SFAs influence endothelial function through modulation of asymmetric dimethylarginine (ADMA), an inhibitor of nitric oxide synthase involved in endothelial dysfunction, which is an early component of atherosclerosis [65,66,67]. Preliminary studies in humans suggest that virgin coconut oil supplementation may reduce fasting glucose and serum triglycerides while improving HDL cholesterol [68], though further research is needed to confirm its clinical benefits in metabolic syndrome (Table 1). 

##### Trans Fatty Acids

**Figure 5:**
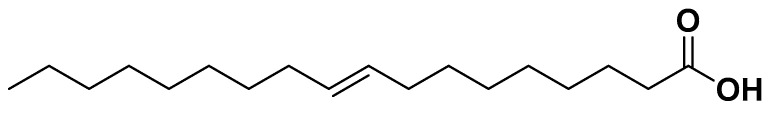
Elaidic acid.

Industrial trans fatty acids like elaidic acid (trans-9-octadecenoic acid) (Figure 5) are consistently associated with adverse metabolic effects, including dyslipidemia, insulin resistance, and increased cardiovascular risk. Their consumption should be minimized to less than 10% of total daily calories [60]. Trans fats exacerbate inflammation and endothelial dysfunction, contributing to the progression of metabolic syndrome (Table 1).

##### Monounsaturated Fatty Acids

**Figure 6:**
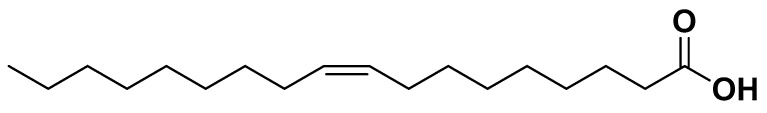
Oleic acid.

Monounsaturated fatty acids (MUFAs), particularly plant-derived oleic acid (Figure 6), are associated with improved lipid profiles, enhanced insulin sensitivity, and reduced cardiovascular risk. Mechanistically, MUFAs modulate gene expression related to lipid metabolism and activate peroxisome proliferator-activated receptor alpha (PPARα), promoting fatty acid β-oxidation and reducing triglyceride synthesis [69]. Epidemiological studies indicate that MUFA-rich diets improve glycemic control and support body weight management (Table 1).

##### Polyunsaturated Fatty Acids

**Figure 7:**
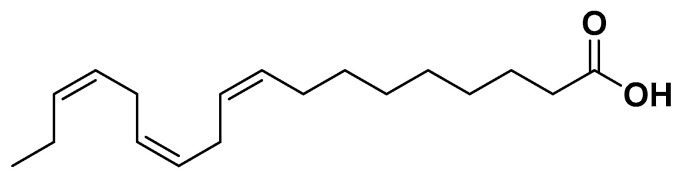
α-linolenic acid (ω-3).

**Figure 8:**
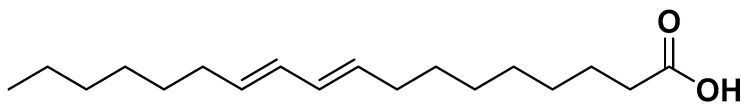
Conjugated linoleic acid.

Polyunsaturated fatty acids (PUFAs), especially omega-3 (α-linolenic acid, Figure 7, eicosapentaenoic acid, docosahexaenoic acid) and omega-6 (linoleic acid, Figure 8) fatty acids, exert multiple beneficial effects on metabolic syndrome components [70,71].

Omega-3 PUFAs improve insulin sensitivity by modulating cell membrane fluidity, activating AMP-activated protein kinase (AMPK), and suppressing hepatic gluconeogenesis. They also reduce chronic low-grade inflammation by downregulating pro-inflammatory cytokines (TNF-α, IL-6) and upregulating anti-inflammatory mediators. Omega-3 PUFAs additionally enhance lipid metabolism by decreasing triglyceride synthesis, promoting fatty acid oxidation, and raising HDL cholesterol levels. They support endothelial function, reduce vascular stiffness, and contribute to blood pressure regulation. Observational studies in populations with high fish consumption, such as the Japanese, demonstrate reduced incidence of type 2 diabetes mellitus and cardiovascular events [71,72,73]. Conjugated linoleic acid (cis-9-trans-11 isomer, Figure 8) has also been reported to improve metabolic markers and insulin sensitivity in adipose tissue and liver [71] (Table 1).

### 4.2. Vitamins

Vitamins play essential roles in metabolic regulation, redox balance, inflammation, and endocrine signaling. However, not all vitamins have demonstrated consistent or clinically relevant effects on the components of metabolic syndrome. Therefore, this review focuses on selected vitamins (A, B2, B6, B7, C, D, E, and K) for which mechanistic data and experimental or clinical evidence suggest a plausible role in modulating insulin resistance, dyslipidemia, oxidative stress, inflammation, or vascular dysfunction. Vitamins with limited or inconsistent evidence in relation to metabolic syndrome were excluded to maintain scientific rigor and clinical relevance [73,74].

#### 4.2.1. Vitamina A

##### Mechanism

**Figure 9:**
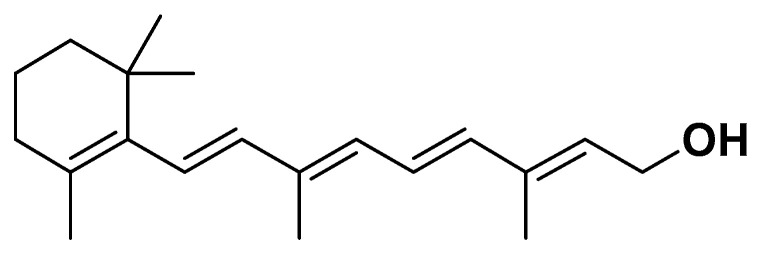
Vitamin A.

Vitamin A, containing a β-ionone ring linked to an isoprenoid chain (Figure 9) and its precursors such as beta-carotene play important roles in metabolic homeostasis and may influence the development and progression of metabolic syndrome.

Vitamin A and its active metabolites, retinoids, regulate gene expression through retinoic acid receptors (RARs) and retinoid X receptors (RXRs), modulating pathways involved in adipogenesis, lipid metabolism, and glucose homeostasis. Beta-carotene, a major dietary provitamin A carotenoid, exhibits antioxidant properties, scavenging reactive oxygen species and reducing oxidative stress as a key contributor to insulin resistance and endothelial dysfunction in MetS. Retinoids also influence adipokine secretion, reducing pro-inflammatory cytokines and improving insulin sensitivity. Epidemiological studies have shown inverse associations between serum beta-carotene levels and components of MetS, including central obesity, dyslipidemia, and elevated blood glucose.

##### Clinical and Metabolic Effects

Antioxidant compounds can modulate oxidative stress and prevent its associated complications [74]. For instance, vitamin A in the form of retinol, according to data obtained in vitro and in vivo studies, not only influences immune function but is also able to reverse chronic inflammation by reducing adipokine levels. Furthermore, data from animal studies indicate that retinol-binding protein “RBP4” concentrations are inversely related to insulin sensitivity, while data from studies on obese or diabetic patients have identified an increase in serum RBP4 levels [75] (Table 1).

#### 4.2.2. Vitamina B2

##### Mechanism

**Figure 10:**
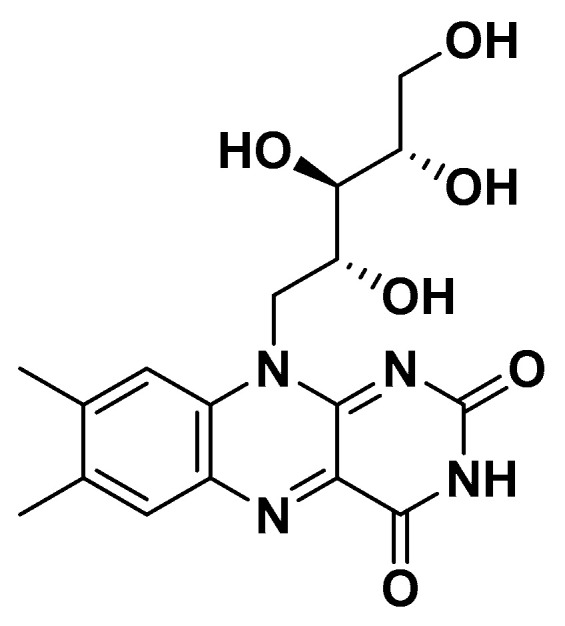
Vitamin B2.

Vitamin B2 (Figure 10), or riboflavin, is as a precursor for the coenzymes flavin mononucleotide (FMN) and flavin adenine dinucleotide (FAD), which are essential for redox reactions in energy metabolism, including the electron transport chain, fatty acid oxidation, and amino acid catabolism. Through these coenzymes, riboflavin regulates cellular energy production, reduces oxidative stress, and maintains proper mitochondrial function, all of which are critical in metabolic syndrome.

##### Clinical and Metabolic Effects

Low riboflavin status has been associated with impaired mitochondrial function, increased oxidative stress, and altered lipid metabolism. Although direct randomized controlled trials (RCTs) in metabolic syndrome are limited, riboflavin deficiency may exacerbate insulin resistance and endothelial dysfunction, supporting its biological relevance. Deficiency in riboflavin has been associated with elevated plasma homocysteine, impaired lipid metabolism, and increased oxidative stress factors that contribute to the pathophysiology of metabolic syndrome (Table 1).

#### 4.2.3. Vitamina B3

##### Mechanism

**Figure 11:**
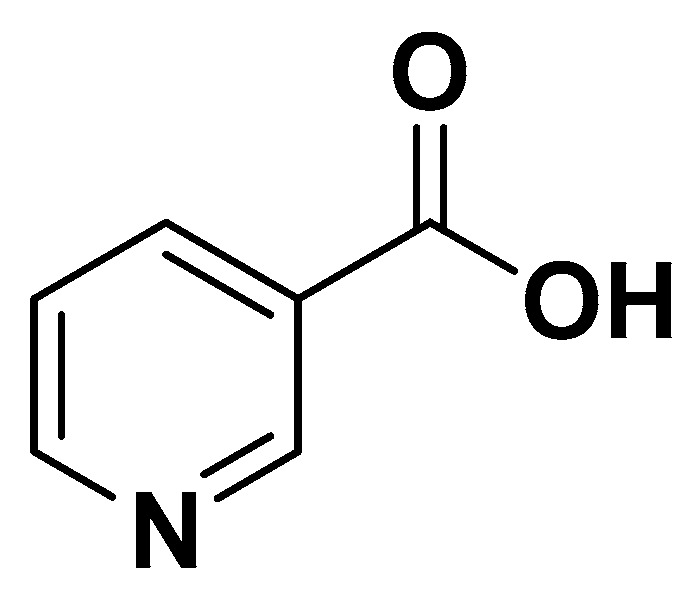
Vitamina B3.

Vitamin B3 or niacin (Figure 11) is a precursor of nicotinamide adenine dinucleotide (NAD^+^) and NAD phosphate (NADP^+^), which are essential cofactors for redox reactions, mitochondrial energy metabolism, and cellular signaling. Pharmacologically, niacin activates the G protein-coupled receptor GPR109A (HM74A) in adipose tissue, inhibiting hormone-sensitive lipase and reducing free fatty acid flux to the liver. This mechanism leads to decreased hepatic triglyceride synthesis and very-low-density lipoprotein (VLDL) production.

##### Clinical and Metabolic Effects

In the context of metabolic syndrome, niacin exerts multiple beneficial mechanisms. It improves lipid metabolism by reducing plasma triglycerides, decreasing low-density lipoprotein cholesterol (LDL-C), and increasing high-density lipoprotein cholesterol (HDL-C). Niacin also modulates inflammatory pathways, lowering pro-inflammatory cytokines and improving endothelial function (Table 1).

#### 4.2.4. Vitamina B6

##### Mechanism

**Figure 12:**
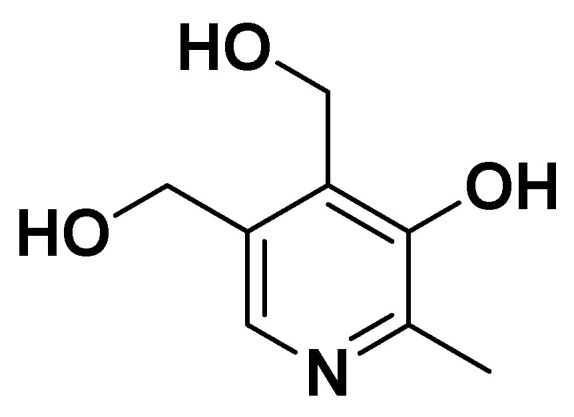
Vitamin B6.

Vitamin B6, or pyridoxine (Figure 12), serves as a coenzyme for over 100 enzymatic reactions, particularly in amino acid metabolism, neurotransmitter synthesis, and one-carbon metabolism. Mechanistically, pyridoxal phosphate, the active form of B6, contributes to glucose homeostasis and modulates inflammatory pathways by reducing pro-inflammatory cytokines such as TNF-α and IL-6.

##### Clinical and Metabolic Effects

It is involved in amino acid metabolism, homocysteine regulation, and neurotransmitter synthesis. It modulates inflammation through effects on cytokine production and supports glucose metabolism via glycogen phosphorylase activity. In metabolic syndrome, vitamin B6 plays a key role in regulating homocysteine metabolism, converting homocysteine to cysteine via the transsulfuration pathway, which reduces cardiovascular risk and endothelial dysfunction. Deficiency in vitamin B6 is associated with insulin resistance, dyslipidemia, and chronic inflammation. Low plasma vitamin B6 levels are associated with obesity, chronic inflammation, and increased cardiovascular risk (Table 1).

#### 4.2.5. Vitamin B7

##### Mechanism

**Figure 13:**
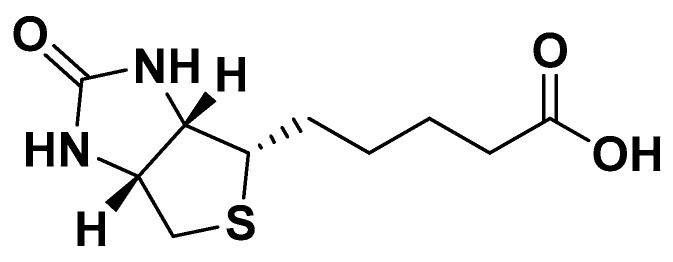
Vitamina B7.

Biotin, also known as vitamin B7 (Figure 13), is important as a coenzyme for carboxylases involved in key metabolic pathways, including gluconeogenesis, fatty acid synthesis, and amino acid catabolism.

##### Clinical and Metabolic Effects

Through its role in these enzymatic reactions, biotin contributes to the regulation of glucose and lipid metabolism, processes that are often dysregulated in metabolic syndrome. Clearly, the biotin deficiency can lead to impaired insulin secretion, hyperglycemia, and dyslipidemia, suggesting a potential link between low biotin status and the development of metabolic syndrome components. Biotin can modulate gene expression related to glucose transporters and lipid-regulating enzymes, reduce oxidative stress, and attenuate inflammatory responses, which are critical contributors to metabolic syndrome (Table 1).

#### 4.2.6. Vitamin C and E

##### Mechanism

**Figure 14:**
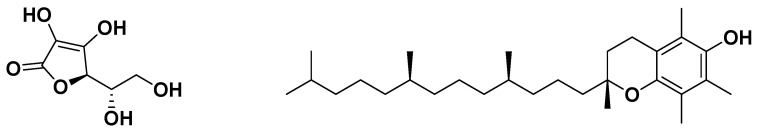
Vitamin C and E, respectively.

Vitamins C (ascorbic acid) and E (tocopherols and tocotrienols) (Figure 14) are key antioxidant vitamins that play complementary roles in mitigating oxidative stress and inflammation. The daily requirement of vitamin C and vitamin E is approximately of 500 mg and 10–15 mg, respectively.

Vitamin C scavenges reactive oxygen species (ROS), regenerates other antioxidants, and supports endothelial function by enhancing nitric oxide bioavailability while Vitamin E protects cell membranes from lipid peroxidation and modulates inflammatory signaling pathways, including NF-κB. Tocotrienols may additionally influence cholesterol synthesis via HMG-CoA reductase inhibition.

##### Clinical and Metabolic Effects

Epidemiological studies have found that diets rich in vitamin C and vitamin E (Figure 6) have positive effects on glucose metabolism, diabetes prevention [74] and cardiovascular risk reduction [73]. In addition, natural forms of these vitamins are more effective than synthetic forms; however, there are still conflicting results on the role of single antioxidants or combinations of various antioxidants in metabolic health [73,74,75,76] (Table 1).

#### 4.2.7. Vitamin D

##### Mechanism

**Figure 15:**
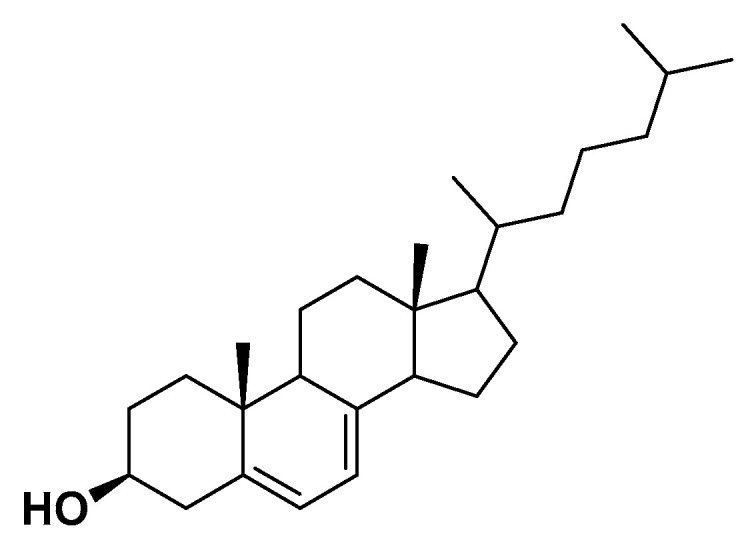
Vitamin D.

Vitamin D (Figure 15) is primarily involved in the regulation of calcium and phosphate homeostasis. However, beyond its role in bone metabolism, growing evidence indicate its involvement in glucose metabolism, blood pressure regulation, and other metabolic parameters related to metabolic syndrome [77]. Vitamin D is mainly synthesized in the skin through the exposure to ultraviolet radiation. It is subsequently metabolized in the liver by the enzyme 25-hydroxylase to form 25-hydroxyvitamin D, and then in the kidneys by the enzyme 1-α-hydroxylase to produce calcitriol, the biologically active form of vitamin D [78]. Calcitriol remains active for up to 27 h and circulating levels of 25-hydroxyvitamin D are generally used as a biomarker for assessing vitamin D status [79,80].

##### Clinical and Metabolic Effects

Specifically, serum concentrations of 25-hydroxyvitamin D ≤ 30 nmol/L indicate a deficient status, concentrations between 30 and 50 nmol/L suggest insufficiency, and levels ranging from 50 to 75 nmol/L are considered adequate [81]. More recently, the relationship between 25-hydroxyvitamin D levels and the risk of metabolic syndrome has garnered interest. A dose–response meta-analysis of 16 cross-sectional studies demonstrated a significant linear inverse association between the two variables [82]. In the context of metabolic syndrome, obese individuals show a decrease in 25-hydroxyvitamin D levels of approximately 0.27 ng/mL for each 1 kg/m^2^ increase in BMI [83]. Among the various hypotheses proposed [84], the concept of reverse causality should be considered: if there is an inverse association between obesity and vitamin D levels, vitamin D may be sequestered or stored in adipose tissue, thereby reducing circulating 25-hydroxyvitamin D concentrations. Therefore, obesity may be a cause, rather than a consequence, of vitamin D deficiency [85]. An inverse association has also been observed between 25-hydroxyvitamin D levels and the incidence of type 2 diabetes mellitus. In a pooled analysis of 21 prospective studies, a relative risk of 0.62 was found for the development of type 2 diabetes [86]. However, randomized controlled trials investigating vitamin D supplementation did not show any significant effect on fasting glucose, HbA1c, insulin resistance, or diabetes incidence. Vitamin D may exert a direct effect on pancreatic β-cell function via activation of the vitamin D receptor expressed on these cells. In contrast, deficiency or absence of this receptor is associated with impaired glucose metabolism [87]. Consequently, vitamin D supplementation is not currently recommended for the prevention or treatment of diabetes due to insufficient supporting evidence [88]. Regarding the association between vitamin D and blood pressure, initial evidence came from observations that low ultraviolet exposure was associated with increased hypertension risk [89]. A meta-analysis involving 283,537 participants examined the relationship between vitamin D status and hypertension risk. Specifically, a 10 ng/mL increase in 25-hydroxyvitamin D levels was associated with a 12% reduction in hypertension risk [90]. Conversely, individuals with levels < 15 ng/mL had a 3.18-fold higher risk of developing hypertension [91]. The antihypertensive effects of vitamin D are believed to be mediated either through direct activation of the vitamin D receptor on vascular smooth muscle cells or through modulation of the renin–angiotensin–aldosterone system [92,93]. By contrast, when considering vitamin D supplementation as a potential intervention to lower blood pressure, the findings have been inconclusive, with no significant reductions in blood pressure observed [94].

Regarding folate and biotin, epidemiological data have linked folate deficiency to an increased risk and incidence of cardiovascular diseases. Additionally, serum folate levels tend to be lower in overweight and obese individuals, suggesting a potential association between folate status and adiposity. Some in vitro studies have reported a synergistic effect between folate and biotin in suppressing pro-inflammatory cytokines [75]; however, relevant human data are currently lacking (Table 1).

#### 4.2.8. Vitamin K

##### Mechanism

**Figure 16:**
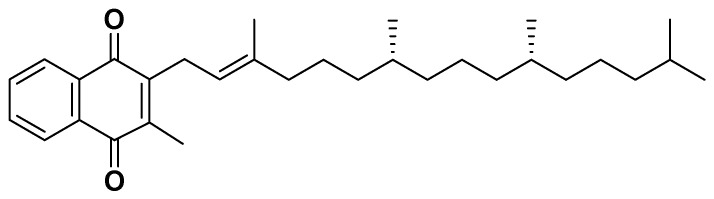
Vitamin K.

Vitamin K (Figure 16) exists primarily as phylloquinone (K1) in green leafy vegetables and menaquinone (K2) in fermented foods and animal products. It plays a critical role in the γ-carboxylation of vitamin K dependent proteins, which are involved in blood coagulation, bone metabolism, and vascular health.

##### Clinical and Metabolic Effects

Emerging evidence indicates that vitamin K also influences glucose and lipid metabolism, suggesting a protective role against components of metabolic syndrome. Observational studies have reported inverse associations between dietary vitamin K intake and insulin resistance, inflammation, and risk of type 2 diabetes mellitus (Table 1).

### 4.3. Polyphenols

**Figure 17:**
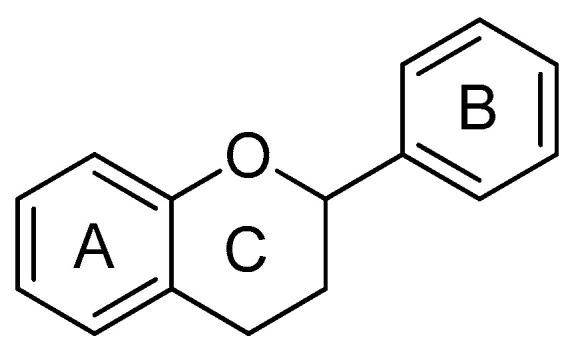
General structure of a flavonoid.

Flavonoids (Figure 17) are a class of naturally occurring polyphenolic compounds characterized by a C6–C3–C6 carbon skeleton, consisting of two aromatic benzene rings (A and B) linked by a three-carbon bridge that usually forms a heterocyclic pyran ring (C ring). They are commonly found in fruits, vegetables, legumes, herbs, and tea. They have been extensively studied due to their anti-inflammatory, antioxidant and antiparasitic properties [95,96,97]. Polyphenols act as antioxidants, scavenging reactive oxygen species (ROS) and reducing oxidative stress, which is a key contributor to insulin resistance, endothelial dysfunction, and chronic inflammation. They also modulate cell signaling pathways, including AMP-activated protein kinase (AMPK), NF-κB, and PPARs, resulting in improved glucose uptake, lipid metabolism, and energy homeostasis. Prospective studies demonstrated an inverse relationship between flavonoid intake and both the incidence and mortality of cardiovascular diseases. Similarly, a systematic review reported that flavonoid-rich foods such as cocoa, chocolate, red wine, grapes and black tea may exert beneficial effects on cardiovascular parameters, including reductions in blood pressure and improvements in endothelial function [73] (Table 1).

**Figure 18:**
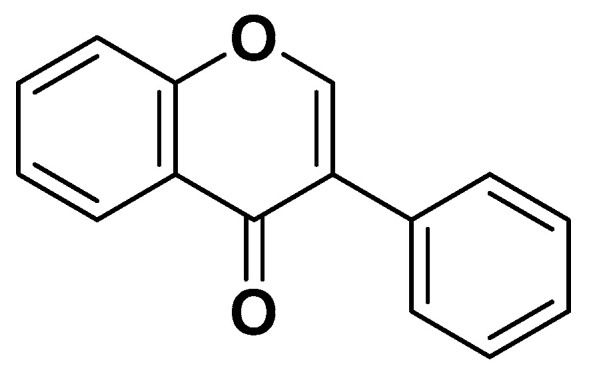
Isoflavone typical of soy.

Soy isoflavones (Figure 18) are a class of polyphenols capable of mimicking the effects of estradiol, thus acting as “phytoestrogens.” Animal studies have shown that soy isoflavones can reduce adiposity and enhance insulin sensitivity; however, evidence in humans remains limited and inconclusive [73].

**Figure 19:**
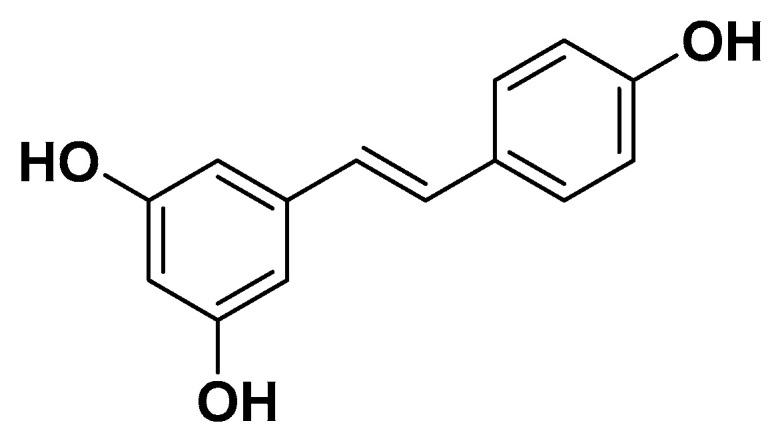
Resveratrol.

Resveratrol (Figure 19) is a naturally occurring polyphenolic stilbene compound chemically defined as 3,5,4′-trihydroxy-trans-stilbene. It is present in various fruits, including grapes, berries, and plums. It is believed to exert potential cardiovascular benefits due to its antioxidant properties and its ability to modulate nitric oxide (NO) levels in the body [72]. Moreover, daily intake of resveratrol at doses of 10 mg or 100 mg has been associated with reductions in LDL cholesterol and improvements in endothelial cell function [76]. More recently, trans-resveratrol supplementation at a dose of 150 mg per day for one month has also been linked to enhanced glucose homeostasis, particularly through improved insulin sensitivity [74]. In animal studies, resveratrol has been shown to reduce fat mass by inhibiting adipocyte differentiation and lipid accumulation, as well as by modulating brown adipose tissue activity, thereby improving energy efficiency. However, further research is needed to clarify its role in weight management in humans (Table 1).

**Figure 20:**
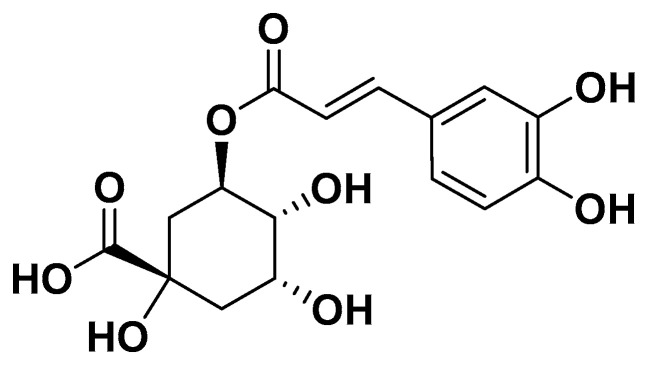
Chlorogenic acid.

Chlorogenic acid (Figure 20) is a naturally occurring polyphenolic ester chemically defined as an ester of caffeic acid and quinic acid. It is widely present in foods such as apples, coffee beans and carrots. This compound, along with its derivatives, has been extensively studied both in vivo and in human subjects to evaluate its role in the prevention and management of metabolic syndrome and related disorders.

Specifically, research involving the administration of either pure chlorogenic acid or foods and supplements rich in this compound has demonstrated beneficial effects on weight management, type 2 diabetes mellitus and hypertension. For instance, a 12-week intake of nutraceuticals containing chlorogenic acid (45 mg) resulted in a weight reduction of 5.4 kg among overweight individuals. Similarly, consumption of a chlorogenic acid-rich beverage (329 mg per 185 mL) over four weeks led to increased postprandial energy expenditure and enhanced fat utilization in healthy subjects. The intake of caffeinated (40 mg/g) and decaffeinated (30 mg/g) coffee contributed to a reduction in glucose-dependent insulinotropic polypeptide levels, thereby decreasing intestinal glucose absorption. Finally, consumption of pure green coffee extract, rich in chlorogenic acid, showed antihypertensive effects in subjects with and without mild hypertension by reducing both systolic and diastolic blood pressure, alongside improvements in endothelial function [97] (Table 1).

**Figure 21:**
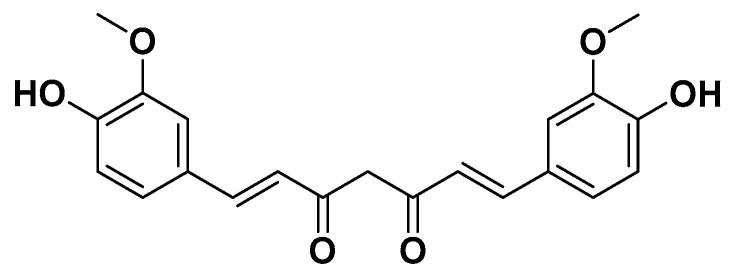
Curcumin.

Curcuminoids are polyphenolic compounds derived from the dried rhizomes of *Curcuma longa* L., responsible for its distinctive yellow-orange coloration [98]. The principal curcuminoid is curcumin, that, together with its structural analogues desmethoxycurcumin and bisdemethoxycurcumin, accounts for approximately 5% of turmeric. Curcumin (Figure 21) is a naturally occurring diarylheptanoid polyphenolic compound chemically defined as a symmetrical diferuloylmethane derivative, IUPAC name 1,7-bis (4-hydroxy-3-methoxyphenyl)-1,6-heptadiene-3,5-dione. It is chemically unstable at physiological pH and rapidly degrades into oxidized products, which are thought to mediate its antioxidant, anti-inflammatory, and hypolipidemic effects [99].

Curcumin exhibits also a broad range of health-promoting properties, alone or in combination [100,101,102,103,104]. Both in vivo and in vitro studies demonstrated its antioxidant and anti-inflammatory activities, specifically, regarding its antioxidant effects, curcumin has been shown to enhance total serum antioxidant capacity and superoxide dismutase activity, increase glutathione levels, and reduce lipid peroxidation. In terms of anti-inflammatory effects, curcumin modulates cytokines, protein kinases, adhesion molecules, and various enzymes. Furthermore, a meta-analysis indicated that curcuminoid supplementation was associated with reduced levels of high-sensitivity C-reactive protein (hs-CRP). Curcumin’s beneficial effects extend to a wide range of conditions, including metabolic syndrome [105], its primary metabolic effect is linked to its insulin-sensitizing activity [106,107], which has been well-documented in diabetic animal models [106] and confirmed in human clinical studies. For metabolic syndrome, the commonly studied daily intake of curcumin is 500–1000 mg per day, usually divided into 1–2 doses. In randomized, double-blind trials, one group of participants received 1500 mg of curcumin daily for nine months, while the control group received a placebo. In prediabetic patients treated with curcumin, all markers of insulin sensitivity, including CRP, HOMA-IR, and HOMA-β, showed significant improvement [108]. However, shorter durations of curcumin treatment did not produce meaningful effects on glucose homeostasis [109]. Moreover, the same study revealed that curcumin supplementation contributed to the prevention of type 2 diabetes mellitus by increasing adiponectin levels by 22.5%. This finding was corroborated by a meta-analysis, which also reported a 26% reduction in leptin levels [101].

A larger study involving 117 individuals with metabolic syndrome confirmed these results, highlighting curcumin’s strong antioxidant and anti-inflammatory actions, as evidenced by significant reductions in CRP levels [110]. Curcumin’s lipid-lowering effect is attributed to multiple mechanisms: it enhances cholesterol efflux through upregulation of ABCA1 and APOA-I expression [111,112], inhibits NPC1L1 expression via SREBP2 [112] and downregulates PCSK9 mRNA expression by 31–48% in various cell lines, thereby promoting LDL receptor expression on cell surfaces and facilitating LDL cholesterol uptake [113].

The downregulation of PCSK9 mRNA is also linked to inhibition of HNF-1α, contributing to curcumin’s anti-inflammatory properties [114]. In murine studies, curcumin has demonstrated anti-atherosclerotic effects by modulating PPAR-α and PPAR-γ receptors, as well as altering CETP and LPL expression key players in fatty acid synthesis and catabolism [115]. Its antioxidant and anti-inflammatory properties, along with its ability to enhance nitric oxide (NO) production [116] are believed to contribute to improved arterial stiffness, which is often exacerbated by inflammation and elevated luminal pressure. For instance, in a study by Alidadi et al., administration of 500 mg of curcumin for 12 weeks in individuals with metabolic syndrome resulted in reductions in body weight and pulse wave velocity (PWV), indicating improved aortic stiffness [117]. Despite its therapeutic potential, curcumin is limited by low oral bioavailability, primarily due to rapid gastrointestinal and hepatic metabolism and poor water solubility. Nonetheless, various formulations have been developed to overcome these barriers: (i) hydrophilic carriers based on cellulose combined with natural antioxidants to enhance absorption [118]; (ii) milk exosome-based encapsulation, which improves resistance to digestion and cellular permeability [119]; (iii) co-formulation with piperine, which inhibits hepatic activity and intestinal glucuronidation (Table 1).

#### Clinical Evidence and Bioavailability Considerations of Curcumin

Clinical trials investigating curcumin supplementation in patients with metabolic syndrome have typically employed doses ranging from 500 to 1500 mg/day, administered either as standard curcumin extracts or enhanced-bioavailability formulations, with intervention durations spanning 8 to 24 weeks. Across randomized controlled trials, curcumin supplementation has shown consistent improvements in inflammatory markers (notably C-reactive protein, TNF-α, and IL-6), reductions in fasting plasma glucose and HOMA-IR, and moderate decreases in triglycerides, while effects on LDL cholesterol and blood pressure appear more variable and less robust. Improvements in waist circumference and body weight have been reported in some studies but are not uniformly observed, suggesting that curcumin’s primary benefits relate more to inflammation control and insulin sensitivity than to direct anthropometric changes [120].

A major limitation of curcumin is its poor oral bioavailability, resulting from low aqueous solubility, rapid intestinal metabolism, and extensive first-pass hepatic clearance. To overcome these pharmacokinetic constraints, several delivery strategies have been developed, including nano-curcumin formulations, phospholipid complexes, micellar systems, and co-administration with bioenhancers such as piperine. Notably, nanocurcumin preparations have demonstrated significantly higher plasma concentrations and enhanced clinical efficacy at lower doses, with improved outcomes on glycemic control, lipid parameters, and systemic inflammation compared to conventional curcumin [120] (Table 1).

### 4.4. Berberine

**Figure 22:**
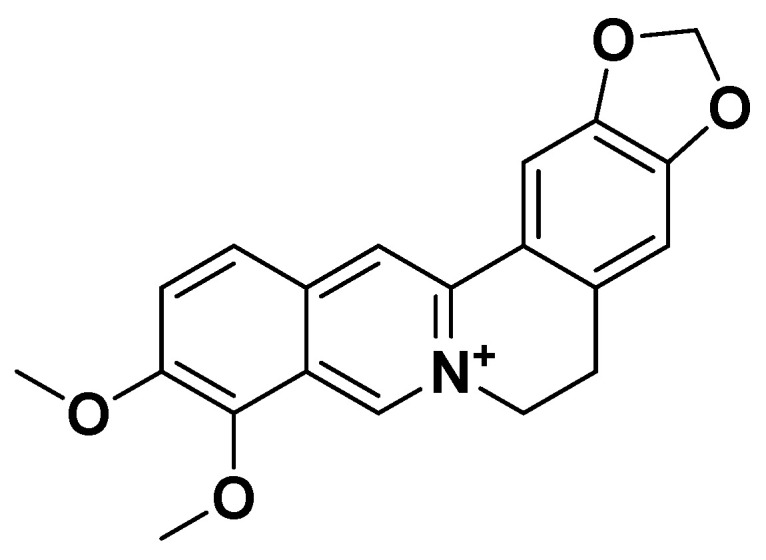
Berberine.

Berberine (Figure 22) is a natural plant-derived isoquinoline alkaloid isolated from the Chinese herb *Coptis chinensis*. Traditionally, it was used in Chinese medicine for its antidiarrheal and hypoglycemic properties. For metabolic syndrome, the typical daily dose of berberine used in clinical studies ranges from 500 mg to 1500 mg per day, usually divided into 2–3 doses taken before meals.

Berberine improves glucose metabolism by activating AMP-activated protein kinase (AMPK), which enhances insulin sensitivity, promotes glucose uptake in skeletal muscle, and suppresses hepatic gluconeogenesis. Berberine also exerts lipid-lowering effects, reducing triglycerides, total cholesterol, and low-density lipoprotein cholesterol (LDL-C) by modulating lipid synthesis and increasing LDL receptor expression. Berberine has been extensively investigated for its metabolic effects, with the strongest and most consistent evidence supporting its role in glycemic control.

More recently, a significant discovery has been its cholesterol-lowering effect in hypercholesterolemic patients [121], which has been attributed to an increase in LDL receptor expression through an ERK-dependent mechanism, independent of sterol regulatory element-binding proteins (SREBPs). Sun et al. [122] demonstrated that berberine not only stabilizes hepatic LDL receptor mRNA via ERK activation but also enhances transcriptional activity of the LDL receptor promoter through the JNK signalling pathway. Moreover, in endothelial cells, berberine was found to counteract the pro-inflammatory and pro-atherogenic effects induced by oxidized LDL and TNF-α, via inhibition of LOX-1 and modulation of AMPK and ERK 1/2 signalling pathways [123]. The mechanisms by which berberine acts on lipid metabolism, particularly those related to metabolic syndrome, have been extensively studied. In the study by Zieniuk et al. [124], berberine inhibited the differentiation of 3T3-L1 adipocytes by modulating transcription factors PPARγ and C/EBPα. Furthermore, it was shown to inhibit PPARα and PPARγ as well as their target genes involved in glucose homeostasis and lipid metabolism [125]. Through these mechanisms, berberine exerts hypolipidemic and hypoglycemic effects and contributes to body weight reduction. In a comparative clinical study, patients with type 2 diabetes were treated with 500 mg of berberine or a similar dose of metformin for 13 weeks. Both treatments led to comparable reductions in HbA1C levels and hypoglycemic effects. Additionally, berberine supplementation resulted in a 21% decrease in triglyceride levels [126]. In the follow-up of the same study, 48 adults with type 2 diabetes exhibited significant reductions in HbA1C, insulin, HOMA index, triglycerides, total cholesterol, and LDL cholesterol. Mbara et al. [127] observed in two animal models of insulin resistance that berberine improved insulin sensitivity and reduced body weight, primarily through AMPK activation in adipocytes and increased GLUT4 translocation in L6 muscle cells. Similarly, in human studies, the administration of 300 mg of berberine for 12 weeks led to significant reductions in BMI, leptin levels, and the HOMA index [128]. Berberine also improves insulin resistance by inhibiting adipose tissue lipolysis. More in detail, its antilipolytic action is associated with increased PDE activity, resulting in reduced cAMP levels and inhibition of HSL activation [129]. Additional studies have shown that berberine, when used in combination with lipid-lowering drugs, has a synergistic effect. Notably, berberine decreases hepatic expression of PCSK9, a protein responsible for LDL receptor degradation [130]. This mechanism is further enhanced when berberine is combined with statins [131]. The reduction in PCSK9 mRNA expression [132] appears to be associated with downregulation of HNF-1α, a key transcriptional cofactor regulating PCSK9 expression [133,134]. When compared with first-line pharmacological agents, the magnitude of berberine’s glycemic effect is broadly comparable to metformin in mild to moderate type 2 diabetes, although direct head-to-head trials remain limited and heterogeneous in design. Lipid-lowering efficacy, while clinically meaningful, is generally inferior to statins but may be relevant in statin intolerant individuals or as adjunctive therapy. Importantly, variability in formulations, dosages, and bioavailability, as well as gastrointestinal tolerability, limits direct quantitative comparisons with standard pharmacotherapy [135]. Therefore, while berberine represents a promising adjunctive nutraceutical for metabolic syndrome particularly for dysglycemia and dyslipidemia its effects should not be overstated as a replacement for established pharmacologic treatments, and further standardized, long-term trials are required to define its optimal clinical positioning. The clinical use of berberine is limited by poor bioavailability, as it is a substrate of P-glycoprotein. As such, higher doses are often required, which may lead to gastrointestinal side effects [136] (Table 1).

### 4.5. Red Yeast Rice

**Figure 23:**
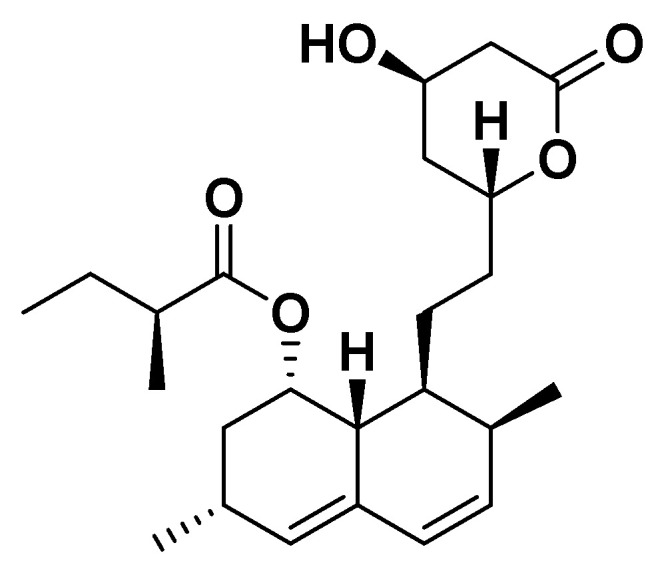
Monacolin K.

Red yeast rice is produced through the fermentation of rice by the yeast *Monascus purpureus*. This fermentation process enhances the rice with various bioactive compounds, including several monacolins (K, M, L, J, and X), among these, monacolin K is the most prominent, possessing a chemical structure like that of lovastatin. Chemically, Monacolin K exists as a lactone prodrug, which is hydrolyzed in vivo to its active β-hydroxy acid form. The molecule features a hexahydronaphthalene (decalin) ring fused to a six-membered lactone ring and a methylbutyrate side chain, providing the structural framework for competitive inhibition of 3-hydroxy-3-methylglutaryl coenzyme A (HMG-CoA) reductase, thus reducing hepatic cholesterol synthesis, and upregulating LDL receptors, which leads to a decreased plasma LDL cholesterol and total cholesterol levels. Consequently, the U.S. Food and Drug Administration (FDA) classifies it as a pharmaceutical agent [137,138]. In contrast, the European Food Safety Authority (EFSA) permits the use of monacolin K (Figure 23) as a nutraceutical for lowering elevated levels of LDL cholesterol [139]. The typical dose of monacolin K is of 10 mg per day, usually taken once daily with meals to improve absorption and reduce gastrointestinal discomfort.

In addition to its cholesterol-lowering properties, red yeast rice has also demonstrated beneficial vascular effects. For instance, a study involving 50 patients with coronary artery disease showed that daily supplementation with 1200 mg of red yeast rice over six weeks resulted in a reduction in fasting high-sensitivity C-reactive protein (hs-CRP) and postprandial flow-mediated dilation (FMD), indicating improved endothelial protection [140]. In a separate study by Cicero et al. involving 40 moderately hypercholesterolemic subjects, administration of 10 mg of monacolin K for four weeks led to enhanced endothelial function, evidenced by a 6% increase in pulse volume (PV) and a 4.7% decrease in pulse wave velocity (PWV) [141]. These findings may be associated with a reduction in plasma concentrations of matrix metalloproteinases MMP-2 and MMP-9 [142]. In rodent models, these vascular effects of red yeast rice were linked to upregulation of endothelial nitric oxide synthase (eNOS) expression and inhibition of oxidative stress in vascular endothelium [143]. However, evidence on the blood pressure-lowering effects of red yeast rice remains limited. In one small, randomized study involving 50 individuals with metabolic syndrome, a combination of 10 mg of monacolins and 9 mg of hydroxytyrosol administered for eight weeks led to reductions in systolic and diastolic blood pressure by 10 mmHg and 7 mmHg, respectively [144]. Currently, red yeast rice is often used in combination with other nutraceuticals to enhance cholesterol-lowering effects while minimizing dosage. For example, its combination with berberine has proven effective in lowering triglycerides, LDL cholesterol, total cholesterol, and glucose levels, while simultaneously increasing HDL cholesterol levels [145]. Additionally, this combination has shown to improve endothelial function and reduce PWV [142]. Unlike statins [146], the red yeast rice and berberine combination also improved the leptin/adiponectin ratio without altering adiponectin levels [147]. Moreover, a nutraceutical formulation consisting of 200 mg of red yeast rice, 500 mg of berberine, and 10 mg of policosanols was shown to improve insulin sensitivity in individuals with insulin resistance, as evidenced by a 24% reduction in HOMA-IR [148]. Finally, a large-scale study involving 2704 hypertensive patients found that red yeast rice supplementation was associated with a reduction in the risk of coronary events and all-cause mortality [140]. Nonetheless, caution is warranted with long-term use of red yeast rice, due to potential increases in creatine kinase (CK) levels and associated myalgia [149,150]. Additionally, poor-quality red yeast rice products may contain citrinin, a nephrotoxic mycotoxin with possible mutagenic and genotoxic effects, as demonstrated in animal models and human lymphocytes [151,152] (Table 1).

### 4.6. Policosanols

**Figure 24:**
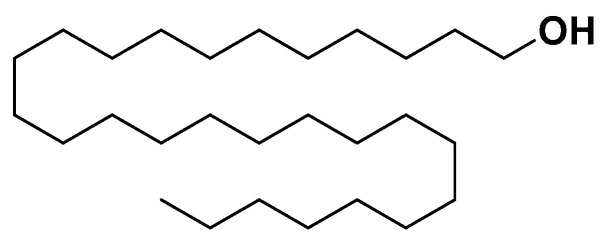
Policosanol.

Policosanols (Figure 24) are a mixture of long-chain aliphatic primary alcohols typically derived from plant waxes, including *Saccharum officinarum* (sugarcane), *Zea mays* (corn), and *Oryza sativa* (rice) bran wax. Chemically, they consist of straight-chain saturated alcohols ranging from C_24_ to C_32_, with octacosanol (C_28_H_58_O) being the most abundant and biologically active component.

They reduce hepatic cholesterol synthesis by partially inhibiting HMG-CoA reductase, like statins but through a milder and safer modulation. A recent meta-analysis of 11 randomized controlled trials involving 3924 hypercholesterolemic patients found that a combination of policosanols, berberine, red yeast rice, folic acid, coenzyme Q10, and astaxanthin led to reductions in total cholesterol, LDL cholesterol, HDL cholesterol, and triglyceride levels [72,153,154] (Table 1).

## 5. Clinical Relevance and Minimum Clinically Important Difference (MCID) Across Nutraceutical Interventions

A major limitation of nutraceutical research in metabolic syndrome is the frequent reliance on statistical significance without adequate consideration of clinical relevance, as defined by the Minimum Clinically Important Difference (MCID). MCID represents the smallest change in a clinical parameter that is likely to translate into a meaningful reduction in cardiometabolic risk or to influence clinical decision-making rather than merely reflecting biological variability or measurement sensitivity [154].

For the core components of metabolic syndrome, clinically meaningful thresholds have been proposed in clinical guidelines and outcome-based studies, including reductions in fasting plasma glucose of approximately 10–15 mg/dL, HbA1c of 0.3–0.5%, triglycerides of 20–30 mg/dL, LDL cholesterol of 10–15%, systolic blood pressure of ≥5 mmHg, and body weight reductions of ≥5% of baseline [155].

Plant proteins such as lupin and soy are associated with modest reductions in LDL cholesterol and postprandial glycemia, which in some studies approach MCID thresholds, particularly when animal protein is replaced by plant sources [156].

Saturated fatty acids generally show neutral or detrimental effects, whereas monounsaturated fatty acids and polyunsaturated fatty acids particularly omega-3 fatty acids more consistently achieve MCID relevant reductions in triglycerides, although effects on glycemic control and body weight are typically modest when used alone [60].

For vitamins (A, B2, B3, B6, B7, C, D, E, and K), most studies report statistically significant changes in surrogate metabolic or inflammatory markers; however, clinically meaningful effects on metabolic syndrome components rarely reach MCID thresholds, with notable exceptions including niacin induced lipid modulation and vitamin D supplementation in deficient individuals. Polyphenols including flavonoids, resveratrol, chlorogenic acid, and curcumin show consistent antioxidant and anti inflammatory effects and modest improvements in insulin sensitivity. Nevertheless, translation to MCID relevant changes in glycemia, lipids, or blood pressure remains inconsistent, largely due to limited bioavailability and short intervention durations. Among the nutraceuticals examined, berberine and red yeast rice show the strongest alignment with MCID benchmarks. Overall, this evidence indicates that many nutraceuticals exert statistically significant but clinically modest effects when assessed against MCID criteria. Their primary utility therefore lies in adjunctive strategies, long term prevention, or risk-factor modulation rather than substitution for evidence-based pharmacological therapy. Explicit integration of MCID into nutraceutical evaluation enhances the translational relevance of this review and provides a more clinically meaningful framework for interpreting future intervention studies.

## 6. Safety and Regulation of Nutraceuticals

Although nutraceuticals such as red yeast rice (RYR) have demonstrated benefits in metabolic syndrome, their clinical use is limited by safety concerns and regulatory inconsistencies. A major hazard in RYR is the mycotoxin citrinin [157], produced during fermentation, which exhibits nephrotoxic, hepatotoxic, and genotoxic effects even at low concentrations. The concentration of citrinin can vary widely across commercial products due to non-standardized fermentation methods and poor-quality control, creating unpredictable exposure risks. In addition, RYR products contain monacolin K [158], the bioactive HMG-CoA reductase inhibitor responsible for lipid-lowering effects, but its concentration is often variable and sometimes exceeds pharmacologically active levels, which may cause adverse effects like statins, including hepatotoxicity, myopathy, and interactions with CYP450-metabolized drugs.

Beyond RYR, the broader nutraceutical industry suffers from lack of standardization, inconsistent labeling, and variable purity, which complicates both safety and efficacy assessments. Reports of contamination with heavy metals, pesticides, or undeclared pharmacological agents have been documented in herbal and dietary supplements, emphasizing the need for stringent quality assurance and post-market surveillance. Regulatory frameworks differ globally: for example, in the United States, RYR is often classified as a dietary supplement, subject to less rigorous oversight, whereas the European Food Safety Authority (EFSA) considers monacolin K a pharmaceutical-like substance, requiring specific safety evaluations. To ensure safe clinical application, nutraceuticals should undergo GMP-compliant production, standardized quantification of active ingredients, maximum contaminant limits, toxicological evaluation, and monitoring for drug–nutrient interactions. Incorporating these measures will mitigate risks and facilitate the responsible integration of nutraceuticals into metabolic syndrome management, balancing therapeutic potential with patient safety [159].

## 7. Delivery Strategies for Nutraceuticals

Despite their potent antioxidant, anti-inflammatory, and metabolic regulatory effects, many nutraceuticals, including resveratrol and curcumin, face major pharmacokinetic limitations that reduce their clinical efficacy in metabolic syndrome. These limitations include poor aqueous solubility, chemical instability in the gastrointestinal tract, rapid phase II metabolism (glucuronidation and sulfation), extensive first-pass hepatic clearance, and limited tissue distribution, which collectively result in low systemic bioavailability. To address these challenges, advanced delivery systems have been developed to enhance absorption, protect bioactive compounds from metabolic degradation, and increase cellular uptake.

Nanoformulations, such as liposomes, solid lipid nanoparticles, polymeric nanoparticles, nanomicelles, and nanogels, improve solubility and stability, prolong circulation time, and facilitate targeted intracellular delivery, including to hepatic and adipose tissues involved in MetS. Phytosome or phospholipid complexation enhances intestinal absorption by forming stable molecular complexes with phosphatidylcholine, which increases lipophilicity and membrane permeability. Cyclodextrin inclusion complexes and micelle-based systems protect bioactives from acidic gastric environments and enzymatic degradation. Furthermore, bioenhancers, such as piperine, quercetin, and other flavonoids, inhibit metabolizing enzymes (e.g., CYP450 isoforms, UDP-glucuronosyltransferases), reduce first pass metabolism, and significantly increase plasma concentrations and half-life of these compounds.

These approaches not only improve systemic and tissue bioavailability but also allow for enhanced modulation of key metabolic and signaling pathways, including AMPK activation, NF-κB inhibition, and PPARα/γ regulation, which underlie the beneficial effects of nutraceuticals on oxidative stress, inflammation, glucose homeostasis, lipid metabolism, and endothelial function. Preclinical and clinical studies demonstrate that such delivery strategies result in greater efficacy at lower doses, improved patient compliance, and reduced interindividual variability. Future research integrating formulation optimization, pharmacokinetics, and clinical validation is essential to translate these compounds into effective nutraceutical therapies for metabolic syndrome [160].

## 8. Conclusions

Metabolic syndrome is a multifactorial and complex clinical condition characterized by a cluster of metabolic disturbances, including abdominal obesity, insulin resistance, dyslipidemia, and hypertension. Despite extensive research, the underlying pathophysiological mechanisms of metabolic syndrome remain incompletely understood, particularly regarding the interplay between genetic, environmental and lifestyle factors. Emerging evidence suggests that chronic low-grade inflammation, oxidative stress, adipokine dysregulation, and endothelial dysfunction all contribute to the onset and progression of this condition, highlighting the need for an integrative approach to its study and management.

Alarmingly, metabolic syndrome is exhibiting a progressive global increase in prevalence, affecting both pediatric and adult populations. This trend is particularly pronounced in populations experiencing lifestyle transitions, urbanization and increased caloric intake, compounded by sedentary behavior. The rising prevalence not only poses a significant health challenge due to its association with type 2 diabetes mellitus, cardiovascular diseases and other comorbidities but also represents a substantial economic burden. The direct and indirect costs related to medical care, pharmacological treatments, hospitalization and loss of productivity are increasingly significant for healthcare systems worldwide.

Given these challenges, elucidating the intricate pathophysiological processes underlying metabolic syndrome is a primary focus of contemporary research. A comprehensive understanding of these mechanisms is essential for the development of more effective, targeted and personalized therapeutic strategies. However, despite advances in pharmacological and clinical interventions, prevention remains a cornerstone in mitigating the onset and progression of metabolic syndrome and its associated complications.

Preventive strategies should ideally begin early in life, emphasizing the modification of lifestyle habits. Key measures include regular physical activity, maintenance of a healthy body weight, and adherence to a nutritionally balanced, health-promoting diet. Within this preventive framework, functional foods and nutraceuticals are increasingly recognized as valuable adjuncts, offering potential benefits in modulating glucose and lipid metabolism, reducing oxidative stress and inflammation and supporting cardiovascular health.

Evidence from clinical and preclinical studies suggests that these bioactive compounds, when combined with conventional dietary and lifestyle interventions, may provide synergistic effects that enhance metabolic homeostasis and reduce the long-term risk of chronic diseases associated with metabolic syndrome.

Overall, addressing metabolic syndrome requires a multifaceted approach that integrates lifestyle modification, dietary strategies, and when appropriate, nutraceutical interventions. Such a comprehensive approach not only targets the underlying pathophysiology of the syndrome but also supports long-term health outcomes, emphasizing the importance of prevention and early intervention as integral components of global health strategies.

## Figures and Tables

**Table 1:** Practical considerations for selected nutraceuticals in the management of metabolic syndrome.

Nutraceutical	Drug–Supplement Interactions	Contraindications and Cautions	Safety Monitoring
Lupin proteins	Additive LDL-cholesterol reduction with statins; potential interaction with antidiabetic drugs	Legume allergy; caution in chronic kidney disease	Lipid profile; fasting glucose; allergy monitoring
Soy proteins	Possible potentiation of lipid-lowering therapies; interaction with thyroid hormone replacement	Soy allergy; estrogen-sensitive conditions	Lipid profile; thyroid function in susceptible individuals
Alanine, Leucine, Valine	Possible interference with insulin-sensitizing agents; mTOR pathway modulation	Insulin resistance, advanced T2DM, liver dysfunction	Insulin sensitivity markers; liver enzymes
Saturated fatty acids	Antagonistic effects with lipid-lowering and antihypertensive drugs	Cardiovascular disease; dyslipidemia	Lipid profile; inflammatory markers
Trans fatty acids	Antagonize cardiometabolic pharmacotherapy	Strongly contraindicated in metabolic syndrome	Avoidance recommended
Monounsaturated fatty acids	Generally safe; additive cardiometabolic benefits	No specific contraindications	Lipid profile; body weight
Polyunsaturated fatty acids (ω-3, ω-6)	Potentiation of anticoagulants and antiplatelet drugs	Bleeding disorders; high-dose supplementation	Triglycerides; coagulation parameters
Vitamin A	Interaction with retinoids; increased hepatotoxicity with alcohol	Pregnancy; liver disease	Liver enzymes; serum retinol (long-term use)
Vitamin B2 (Riboflavin)	No clinically relevant interactions	Generally safe	No routine monitoring
Vitamin B3 (Niacin)	Additive hepatotoxicity with statins; interaction with antihypertensives	Liver disease; gout; uncontrolled diabetes	Liver enzymes; uric acid; fasting glucose
Vitamin B6 (Pyridoxine)	Reduced efficacy of levodopa (without carbidopa)	Chronic high-dose supplementation	Neurological assessment
Vitamin B7 (Biotin)	Interference with laboratory immunoassays	Nonspecific	Temporary discontinuation before lab tests
Vitamin C	Increased iron absorption; interaction with chemotherapy agents	Hemochromatosis; nephrolithiasis (high dose)	Renal function (high doses)
Vitamin D	Interaction with thiazide diuretics (hypercalcemia risk)	Hypercalcemia; granulomatous diseases	Serum 25(OH)D; calcium
Vitamin E	Potentiation of anticoagulants	Bleeding disorders	Coagulation parameters (high dose)
Vitamin K	Antagonizes vitamin K antagonists (e.g., warfarin)	Anticoagulant therapy	INR monitoring
Flavonoids	CYP450 modulation affecting drug metabolism	Polypharmacy	No routine monitoring
Resveratrol	Potentiation of anticoagulants; CYP interactions	Bleeding risk at high doses	Coagulation markers
Chlorogenic acid	Interaction with glucose-lowering drugs	Hypoglycemia risk	Fasting glucose
Curcumin	Potentiation of anticoagulants; CYP modulation	Gallbladder disease; bleeding disorders	Liver enzymes; coagulation parameters
Berberine	Additive effects with antidiabetics and statins; CYP3A4 inhibition	Pregnancy; gastrointestinal disorders	Fasting glucose; lipid profile
Red yeast rice	Statin-like interactions; CYP3A4 inhibitors increase toxicity	Liver disease; pregnancy; statin intolerance	Liver enzymes; CK; citrinin contamination
Policosanols	Potentiation of antiplatelet drugs	Bleeding disorders	Lipid profile; coagulation markers

## Data Availability

No new data were created or analyzed in this study.

## Abbreviations

The following abbreviations are used in this manuscript:

AACE	American Association of Clinical Endocrinology
ABCA1	ATP-binding cassette transporter A1
ABCG5	ATP-binding cassette transporter G5
ABCG8	ATP-binding cassette transporter G8
ACLY	ATP-citrate lyase
ADMA	Asymmetric dimethylarginine
AHA/NHLBI	American Heart Association/National Heart, Lung, and Blood Institute
AMPK	AMP-actived protein kinase
APOA-I	Apolipoprotein A-1
ATP	Adenosine triphosphate
BMI	Body Mass Index
cAMP	cyclic AMP
CDC	Center of Disease Control and Prevention
C/EBPα	CCAAT-enhancer-binding proteins α
C/EBPβ	CCAAT-enhancer-binding proteins β
CETP	Cholesterol ester transfer protein
CFU	Colony Forming Units
CK	Creatine Kinase
CRP	C-reactive protein
DNA	Deoxyribonucleic acid
DPP-IV	Dipeptidyl peptidase-IV
EFSA	European Food Safety Authority
EGIR	European Group for the study of Insulin Resistance
eNOS	Nitric oxide synthase.
ERK	Extracellular signal-regulated kinases.
FADH2	Reduced flavin adenine dinucleotide.
FDA	Food and Drug Administration.
FFAR	Free fatty acid receptor
FFAR2	Free fatty acid receptor 2
FFAR3	Free Fatty Acid Receptor 3
FMD	Flow-Mediated Dilatation
FTO	Fat mass and obesity associated
GDF15	Growth differentiation factor 15
GLP-1	Glucagon-like peptide-1
GLUT4	Glucose Transporter 4
GPR21	G protein-coupled receptors 21
GPR82	G protein-coupled receptors 82.
HbA1C	Glycated hemoglobin
HDL	High-density lipoprotein
HSL	Hormone-sensitive lipase
HMGCR	HMG-CoA reductase
HNF-1α	Hepatic nuclear factor -1α
HOMA	Homeostatic model assessment
HOMA-IR	Homeostatic model assesment-insulin resistance
hs-CRP	High sensivity C-reactive protein
IDF	International Diabetes Federation
IL-6	Interleukin-6
Ile-Pro-Pro	Isoleucine–Proline–Proline
IRS-1	Insulin receptor substrate-1
JNK	Jun N-terminal kinase
LDL	Low-density lipoprotein
LOX-1	Lipoprotein receptor-1
LPL	Lipoprotein lipasi
LPS	Lipopolysaccharide
MMP-2	Matrix metalloproteinase-2
MMP-9	Matrix metalloproteinase-9
mRNA	Messenger RNA
NADH	Nicotinamide adenine dinucleotide
NADPH	Nicotinamide adenine dinucleotide phosphate
NCEP	National Cholesterol Education Program
ATPIII	Adult Treatment Panel III
NF- kB	Nuclear factor kappa-light-chain enhancer of activated B cells
NHANES	National Health and Nutrition Examination Survey
NO	Nitric oxide
NPC1L1	Niemann–Pick C1 like 1 protein
NREP	Neuronal regeneration–related protein
PAI-1	Plasminogen activator inhibitor-1
PCSK9	Proprotein convertase subtilisin/kexin type 9
PDE	Phosphodiesterase
PGC-1α	Peroxisome proliferator-activated receptor-γ coactivator -1α
PI3K/Akt	Phosphoinositide 3-kinase/Protein kinase B
PPARα	Peroxisome proliferator-activated receptor α
PPARγ	Peroxisome proliferator-activated receptor γ
PPARδ	Peroxisome proliferator-activated receptor δ
PV	Pulse volume
PWV	Pulse wave velocity
RBP4	Retinol-binding protein 4
RNA	Ribonucleic acid
ROS	Reactive Oxygen Species
SCFA	Short chain fatty acids
SNP	Single nucleotide polymorphisms
SREBP	Sterol regulatory element-binding protein
SREBP-1c	Sterol regulatory element-binding protein-1c
SREBP2	Sterol regulatory element-binding protein 2
TGF-β	Transforming growth factor- β
Th1	T helper type 1
TNFα	Tumor necrosis factor α
Val-Pro-Pro	Valine–Proline–Proline
VLDL	Very low-density lipoprotein
WHO	World Health Organization

## References

[1] Accili D., Deng Z., Liu Q. (2025). Insulin resistance in type 2 diabetes mellitus. Nat. Rev. Endocrinol..

[2] Ferrannini E. (2021). A Journey in Diabetes: From Clinical Physiology to Novel Therapeutics: The 2020 Banting Medal for Scientific Achievement Lecture. Diabetes.

[3] Cheng T.O. (2006). Cardiac syndrome X versus metabolic syndrome X. Int. J. Cardiol..

[4] Alberti K.G., Zimmet P.Z. (1998). Definition, diagnosis and classification of diabetes mellitus and its complications. Part 1: Diagnosis and classification of diabetes mellitus provisional report of a WHO consultation. Diabet. Med..

[5] Balkau B., Charles M.A. (1999). Comment on the provisional report from the WHO consultation. European Group for the Study of Insulin Resistance (EGIR). Diabet. Med..

[6] National Cholesterol Education Program (NCEP) Expert Panel (2001). Executive Summary of The Third Report of The National Cholesterol Education Program (NCEP) Expert Panel on Detection, Evaluation, And Treatment of High Blood Cholesterol In Adults (Adult Treatment Panel III). JAMA.

[7] Ucar E., Huzmeli C., Guven O., Savas N., Gullu M., Asilyoruk S., Kuvandik C., Temizkan A., Kuvandik G. (2009). Frequency of metabolic syndrome among hemodialysis patients according to NCEP-ATP III and IDF definitions. Ren. Fail..

[8] Einhorn D., Reaven G.M., Cobin R.H., Ford E., Ganda O.P., Handelsman Y., Hellman R., Jellinger P.S., Kendall D., Krauss R.M. (2003). American College of Endocrinology position statement on the insulin resistance syndrome. Endocr. Pract..

[9] Alberti K.G., Zimmet P., Shaw J. (2005). The metabolic syndrome–a new worldwide definition. Lancet.

[10] Grundy S.M., Brewer H.B., Cleeman J.I., Smith S.C., Lenfant C. (2004). Definition of metabolic syndrome: Report of the National Heart, Lung, and Blood Institute/American Heart Association conference on scientific issues related to definition. Arterioscler. Thromb. Vasc. Biol..

[11] Mancia G., Bombelli M., Facchetti R., Casati A., Ronchi I., Quarti-Trevano F., Arenare F., Grassi G., Sega R. (2010). Impact of different definitions of the metabolic syndrome on the prevalence of organ damage, cardiometabolic risk and cardiovascular events. J. Hypertens..

[12] Alberti K.G., Eckel R.H., Grundy S.M., Zimmet P.Z., Cleeman J.I., Donato K.A., Fruchart J.C., James W.P., Loria C.M., Smith S.C. (2009). Harmonizing the metabolic syndrome: A joint interim statement of the International Diabetes Federation Task Force on Epidemiology and Prevention; National Heart, Lung, and Blood Institute; American Heart Association; World Heart Federation; International Atherosclerosis Society; and International Association for the Study of Obesity. Circulation.

[13] Williamson G. (2025). Bioavailability of Food Polyphenols: Current State of Knowledge. Annu. Rev. Food Sci. Technol..

[14] Ross R., Neeland I.J., Yamashita S., Shai I., Seidell J., Magni P., Santos R.D., Arsenault B., Cuevas A., Hu F.B. (2020). Waist circumference as a vital sign in clinical practice: A Consensus Statement from the IAS and ICCR Working Group on Visceral Obesity. Nat. Rev. Endocrinol..

[15] Tanasescu M.-D., Rosu A.-M., Minca A., Rosu A.-L., Grigorie M.-M., Timofte D., Ionescu D. (2025). Beyond BMI: Rethinking Obesity Metrics and Cardiovascular Risk in the Era of Precision Medicine. Diagnostics.

[16] Khawaja T., Nied M., Wilgor A., Neeland I.J. (2024). Impact of Visceral and Hepatic Fat on Cardiometabolic Health. Curr. Cardiol. Rep..

[17] Nauli A.M., Matin S. (2019). Why do men accumulate abdominal visceral fat?. Front. Physiol..

[18] Lv Z., Fu Y., Ma Y., Liu C., Yuan M., Gao D. (2025). Associations Between Visceral and Liver Fat and Cardiac Structure and Function: A UK Biobank Study. J. Clin. Endocrinol. Metab..

[19] Saklayen M.G. (2018). The global epidemic of the metabolic syndrome. Curr. Hypertens. Rep..

[20] Gallardo-Alfaro L., Bibiloni M.D.M., Mateos D., Ugarriza L., Tur J.A. (2019). Leisure-Time Physical Activity and Metabolic Syndrome in Older Adults. Int. J. Environ. Res. Public Health.

[21] Noubiap J.J., Nansseu J.R., Lontchi-Yimagou E., Nkeck J.R., Nyaga U.F., Ngouo A.T., Tounouga D.N., Tianyi F.L., Foka A.J., Ndoadoumgue A.L. (2022). Geographic Distribution of Metabolic Syndrome and Its Components in the General Adult Population: A Meta-Analysis of Global Data from 28 million Individuals. Diabetes Res. Clin. Pract..

[22] Zhang Y., Shi J., Cai J., Yang Y., Xiu L. (2025). Metabolic Syndrome in Children and Adolescents: Definitions, Epidemiology, Pathophysiology, Interventions, and Challenges. Front. Endocrinol..

[23] Kelsey M.M., Pyle L., Hilkin A., Severn C.D., Utzschneider K., Van Pelt R.E., Nadeau K.J., Zeitler P.S. (2020). The Impact of Obesity on Insulin Sensitivity and Secretion During Pubertal Progression: A Longitudinal Study. J. Clin. Endocrinol. Metab..

[24] González-Domínguez Á., Domínguez-Riscart J., Savolainen O., Lechuga-Sancho A., Landberg R., González-Domínguez R. (2024). Identifying Metabotypes of Insulin Resistance Severity in Children with Metabolic Syndrome. Cardiovasc. Diabetol..

[25] Jurado-Sumariva L., González-Domínguez Á., Savolainen O., Domínguez-Riscart J., Landberg R., González-Domínguez R. (2025). Insulin Resistance as a Potential Driving Force of Parental Obesity-Induced Adverse Metabolic Programming Mechanisms in Children with Obesity. BMC Med..

[26] Mousavi E., Mozafarian N., Heidari-Beni M., Sehhati M., Kelishadi R. (2025). Uncovering Age-Specific Subtypes of Pediatric Obesity and Metabolic Syndrome Using Machine Learning Algorithms. Sci. Rep..

[27] Kartiosuo N., Raitakari O.T., Juonala M., Viikari J.S.A., Sinaiko A.R., Venn A.J., Jacobs D.R., Urbina E.M., Woo J.G., Steinberger J. (2024). Cardiovascular Risk Factors in Childhood and Adulthood and Cardiovascular Disease in Middle Age. JAMA Netw. Open.

[28] Wu P.-W., Lai Y.-W., Chin Y.-T., Tsai S., Yang T.-M., Lin W.-T., Lee C.-Y., Tsai W.-C., Huang H.-L., Seal D.W. (2022). Stability and Transformation of Metabolic Syndrome in Adolescents: A Prospective Assessment in Relation to the Change of Cardiometabolic Risk Factors. Nutrients.

[29] Chin Y.T., Wu P.W., Huang P.R., Tsai S., Lin W.T., Lee C.Y., Tsai W.-C., Lee C.-H. (2025). Confirmatory-Factor-Analysis-Derived Metabolic Syndrome Risk Score: Development, Validation, and Clinical Utility in Dual Adolescent Populations. Pediatr. Res..

[30] Mira M., Anwar G., Thabet G., Nofal R.E., Hassanein S.A. (2025). Copeptin: A Biochemical Predictor of Metabolic Syndrome in Children and Adolescents with Obesity. Egypt. Pediatr. Assoc. Gaz..

[31] Huang J.S., Zhang X.Y., Ramakrishnan R., Chu J.Q., Lu M.S., Shao D.T., Qiu X., He J.R. (2026). Association of Childhood Obesity Phenotypes With Cardiometabolic Outcomes in Adulthood: A Systematic Review and Meta-Analysis. Obes. Rev..

[32] Żurawski T., Bartosiewicz A. (2026). Dietary and Behavioral Strategies for Weight Loss and Weight Loss Maintenance: A Narrative Review. Nutrients.

[33] Egert S., Amini A.M., Klug L., Kalotai N., Haardt J., Boeing H., Buyken A.E., Kroke A., Lorkowski S., Louis S. (2025). Protein Intake and Cardiovascular Diseases: An Umbrella Review of Systematic Reviews for the Evidence-Based Guideline on Protein Intake of the German Nutrition Society. Eur. J. Nutr..

[34] Pan A., Sun Q., Bernstein A.M., Schulze M.B., Manson J.E., Stampfer M.J., Willett W.C., Hu F.B. (2013). Changes in Red Meat Consumption and Subsequent Risk of Type 2 Diabetes Mellitus: Three Cohorts of US Men and Women. JAMA Intern. Med..

[35] Virtanen H.E.K., Koskinen T.T., Voutilainen S., Mursu J., Tuomainen T.P., Kokko H., Ylilauri M.P.T., Salonen J.T., Virtanen J.K. (2017). Intake of Different Dietary Proteins and Risk of Type 2 Diabetes in Men: The Kuopio Ischaemic Heart Disease Risk Factor Study. Br. J. Nutr..

[36] Kaur J. (2014). A Comprehensive Review on Metabolic Syndrome. Cardiol. Res. Pract..

[37] Arnoldi A., Boschin G., Zanoni C., Lammi C., Zanoni C., Scarafoni A., Sirtori C.R. (2015). The Health Benefits of Sweet Lupin Seed Flours and Isolated Proteins. J. Funct. Foods.

[38] Chen M., Li Y., Liu X. (2025). A Review of the Role of Bioactive Components in Legumes in the Prevention and Treatment of Cardiovascular Diseases. Food Funct..

[39] Bryant L., Rangan A., Grafenauer S. (2022). Lupins and Health Outcomes: A Systematic Literature Review. Nutrients.

[40] Bahr M., Fechner A., Kiehntopf M., Jahreis G. (2015). Consuming a Mixed Diet Enriched with Lupin Protein Beneficially Affects Plasma Lipids in Hypercholesterolemic Subjects: A Randomized Controlled Trial. Clin. Nutr..

[41] Pavanello C., Lammi C., Ruscica M., Bosisio R., Mombelli G., Soverini V., D’Orazio A., Morlotti B., Stragliotto E., Della Vedova F. (2017). Effects of a Lupin Protein Concentrate on Lipids, Blood Pressure and Insulin Resistance in Moderately Dyslipidaemic Patients: A Randomised Controlled Trial. J. Funct. Foods.

[42] Urban D., Pöss J., Böhm M., Laufs U. (2013). Targeting the Proprotein Convertase Subtilisin/Kexin Type 9 for the Treatment of Dyslipidemia and Atherosclerosis. J. Am. Coll. Cardiol..

[43] Ferri N., Ruscica M. (2016). Proprotein Convertase Subtilisin/Kexin Type 9 (PCSK9) and Metabolic Syndrome: Insights on Insulin Resistance, Inflammation, and Atherogenic Dyslipidemia. Endocrine.

[44] Soto-Luna I.C., García-López P.M., Vargas-Guerrero B., Guzmán T.J., Domínguez-Rosales J.A., Gurrola-Díaz C.M. (2021). Lupin Protein Isolate Improves Insulin Sensitivity and Steatohepatitis In Vivo and Modulates the Expression of the *Fasn*, *Gys2*, and *Gsk3b* Genes. Food Sci. Nutr..

[45] Scirica B.M., Bhatt D.L., Braunwald E., Steg P.G., Davidson J., Hirshberg B., Ohman P., Frederich R., Wiviott S.D., Hoffman E.B. (2013). Saxagliptin and Cardiovascular Outcomes in Patients with Type 2 Diabetes Mellitus. N. Engl. J. Med..

[46] Lammi C., Zanoni C., Arnoldi A., Vistoli G. (2016). Peptides Derived from Soy and Lupin Protein as Dipeptidyl-Peptidase IV Inhibitors: In Vitro Biochemical Screening and In Silico Molecular Modeling Study. J. Agric. Food Chem..

[47] Santos-Sánchez G., Cruz-Chamorro I., Álvarez-Ríos A.I., Álvarez-Sánchez N., Rodríguez-Ortiz B., Álvarez-López A.I., Fernández-Pachón M.S., Pedroche J., Millán F., Millán-Linares M.D.C. (2022). Bioactive Peptides from Lupin (*Lupinus angustifolius*) Prevent the Early Stages of Atherosclerosis in Western Diet-Fed *ApoE*–/– Mice. J. Agric. Food Chem..

[48] Chalvon-Demersay T., Azzout-Marniche D., Arfsten J., Egli L., Gaudichon C., Karagounis L.G., Tomé D. (2017). A Systematic Review of the Effects of Plant Compared with Animal Protein Sources on Features of Metabolic Syndrome. J. Nutr..

[49] Zuo X., Zhao R., Wu M., Wan Q., Li T. (2023). Soy Consumption and the Risk of Type 2 Diabetes and Cardiovascular Diseases: A Systematic Review and Meta-Analysis. Nutrients.

[50] Papaodyssea I., Lagiou A., Tzoulaki I., Valanou E., Naska A. (2025). The Effect of Increased Plant Protein Intake on the Lipid Profile of Chronic Kidney Disease Patients: A Meta-Analysis of Controlled Clinical Trials. Nutrients.

[51] Sohouli M.H., Lari A., Fatahi S., Shidfar F., Găman M.-A., Guimarăes N.S., Sindi G.A., Mandili R.A., Alzahrani G.R., Abdulwahab R.A. (2021). Impact of Soy Milk Consumption on Cardiometabolic Risk Factors: A Systematic Review and Meta-Analysis of Randomized Controlled Trials. J. Funct. Foods.

[52] Luo Q., Kang B., Lei L., Yan H., Chen M., Peng T., Ouyang Y., Sun H., Hui S. (2026). Comparative effects of different types of soy products on glycemic control and insulin sensitivity: A network meta-analysis of randomized controlled trials. Front. Nutr..

[53] Vergnaud A.C., Norat T., Mouw T., Romaguera D., May A.M., Bueno-de-Mesquita H.B., van der A D., Agudo A., Wareham N., Khaw K.T. (2013). Macronutrient Composition of the Diet and Prospective Weight Change in Participants of the EPIC-PANACEA Study. PLoS ONE.

[54] Ruscica M., Pavanello C., Gandini S., Macchi C., Botta M., Dall’Orto D., Del Puppo M., Bertolini S., Calabresi L., Sirtori C.R. (2016). Effect of Soy on Metabolic Syndrome and Cardiovascular Risk Factors: A Randomized Controlled Trial. Eur. J. Nutr..

[55] Muñoz-Cabrejas A., Guallar-Castillón P., Laclaustra M., Sandoval-Insausti H., Moreno-Franco B. (2023). Association between Sugar-Sweetened Beverage Consumption and the Risk of the Metabolic Syndrome: A Systematic Review and Meta-Analysis. Nutrients.

[56] Chen H.-P., Kao Y., Lin M.-W., Lee C.-T., Wu H.-T., Kuo H.-Y. (2025). Potential Effects of Low-Calorie Sweeteners on Human Health. Nutrients.

[57] Clifton P. (2019). Metabolic Syndrome—Role of Dietary Fat Type and Quantity. Nutrients.

[58] Kang Y.J., Hwang K.M., Cheon S.Y., Lee H.J., Yoon H.S. (2016). Associations of Obesity and Dyslipidemia with Intake of Sodium, Fat, and Sugar among Koreans: A Qualitative Systematic Review. Clin. Nutr. Res..

[59] Hoyas I., Leon-Sanz M. (2019). Nutritional Challenges in Metabolic Syndrome. J. Clin. Med..

[60] Sacks F.M., Lichtenstein A.H., Wu J.H., Appel L.J., Creager M.A., Kris-Etherton P.M., Miller M., Rimm E.B., Rudel L.L., Robinson J.G. (2017). Dietary Fats and Cardiovascular Disease: A Presidential Advisory from the American Heart Association. Circulation.

[61] Unger A.L., Torres-Gonzalez M., Kraft J. (2019). Dairy Fat Consumption and the Risk of Metabolic Syndrome: An Examination of the Saturated Fatty Acids in Dairy. Nutrients.

[62] Zhang Y., Wang Y., Li L., Hong X., Xia H., Sohouli M.H., Xu Z. (2025). The effect of virgin coconut oil (VCO) on cardiovascular disease risk factors: A systematic review and meta-analysis. Diabetol. Metab. Syndr..

[63] Boateng L., Ansong R., Owusu W., Steiner-Asiedu M. (2016). Coconut Oil and Palm Oil’s Role in Nutrition, Health and National Development: A Review. Ghana Med. J..

[64] Eyres L., Eyres M.F., Chisholm A., Brown R.C. (2016). Coconut Oil Consumption and Cardiovascular Risk Factors in Humans. Nutr. Rev..

[65] Yu C., Zhang M., Xia W. (2025). Asymmetric Dimethylarginine Mediates Oxidative Stress and Atrial Remodeling in HL-1 Cells. Front. Med..

[66] Rakhmat I.I., Nugraha G.I., Ariyanto E.F., Pratiwi Y.S., Linasari D., Fatimah S.N., Ghozali M., Syamsunarno M.R.A.A., Akbar M.R., Achmad T.H. (2024). Strong Association of Metabolic Parameters with ADMA and VCAM-1 in Normo-Weight Subjects with Metabolic Syndrome. Diabetes Metab. Syndr. Obes..

[67] Engeli S., Tsikas D., Lehmann A., Böhnke J., Haas V., Strauß A., Janke J., Gorzelniak K., Luft F., Jordan J. (2012). Influence of Dietary Fat Ingestion on Asymmetrical Dimethylarginine in Lean and Obese Human Subjects. Nutr. Metab. Cardiovasc. Dis..

[68] Nikooei P., Hosseinzadeh-Attar M.J., Asghari S., Norouzy A., Yaseri M., Vasheghani-Farahani A. (2021). Effects of Virgin Coconut Oil Consumption on Metabolic Syndrome Components and Asymmetric Dimethylarginine: A Randomized Controlled Clinical Trial. Nutr. Metab. Cardiovasc. Dis..

[69] Phillips C.M. (2013). Nutrigenetics and Metabolic Disease: Current Status and Implications for Personalised Nutrition. Nutrients.

[70] Calton E.K., James A.P., Pannu P.K., Soares M.J. (2014). Certain Dietary Patterns Are Beneficial for the Metabolic Syndrome: Reviewing the Evidence. Nutr. Res..

[71] Badawy S., Liu Y., Guo M., Liu Z., Xie C., Marawan M.A., Ares I., Lopez-Torres B., Martínez M., Maximiliano J.-E. (2023). Conjugated linoleic acid (CLA) as a functional food: Is it beneficial or not?. Food Res. Int..

[72] Hei Y., Xie Y. (2025). Effects of Exercise Combined with Different Dietary Interventions on Cardiovascular Health: A Systematic Review and Network Meta-Analysis. BMC Cardiovasc. Disord..

[73] Ricottini L., Basciani S., Spizzichini M.L., de Mattia D., Coniglio-Iannuzzi delle Noci M., Sorrentino S., Nordio M. (2024). The Effectiveness and Safety of a Nutraceutical Combination in Overweight Patients with Metabolic Syndrome. Nutrients.

[74] Clemente-Suárez V.J., Martín-Rodríguez A., Beltrán-Velasco A.I., Rubio-Zarapuz A., Martínez-Guardado I., Valcárcel-Martín R., Tornero-Aguilera J.F. (2025). Functional and Therapeutic Roles of Plant-Derived Antioxidants in Type 2 Diabetes Mellitus: Mechanisms, Challenges, and Considerations for Special Populations. Antioxidants.

[75] Dakshinamurti K. (2015). Vitamins and Their Derivatives in the Prevention and Treatment of Metabolic Syndrome Diseases (Diabetes). Can. J. Physiol. Pharmacol..

[76] Xu Y.J., Tappia P.S., Neki N.S., Dhalla N.S. (2014). Prevention of Diabetes-Induced Cardiovascular Complications upon Treatment with Antioxidants. Heart Fail. Rev..

[77] Khan H., Kunutsor S., Franco O.H., Chowdhury R., Chowdhury S., Ernst B., Willeit P., Di Angelantonio E., Danesh J., Forouhi N.G. (2013). Vitamin D, Type 2 Diabetes and Other Metabolic Outcomes: A Systematic Review and Meta-Analysis of Prospective Studies. Proc. Nutr. Soc..

[78] Atef S.H. (2017). Vitamin D Assays in Clinical Laboratory: Past, Present and Future Challenges. J. Steroid Biochem. Mol. Biol..

[79] Holick M.F. (2017). The Vitamin D Deficiency Pandemic: Approaches for Diagnosis, Treatment and Prevention. Rev. Endocr. Metab. Disord..

[80] Sosa-Henríquez M., Torregrosa-Suau Ó., Gómez de Tejada-Romero M.J., Cancelo-Hidalgo M.J., Tarazona-Santabalbina F.J., Etxebarria-Foronda I., Díaz-Guerra G.M., Valdés-Llorca C. (2025). Rethinking Vitamin D Deficiency: Controversies and Practical Guidance for Clinical Management. Nutrients.

[81] Grant W.B., Wimalawansa S.J., Pludowski P., Cheng R.Z. (2025). Vitamin D: Evidence-Based Health Benefits and Recommendations for Population Guidelines. Nutrients.

[82] Lee K., Kim J. (2021). Serum vitamin D status and metabolic syndrome: A systematic review and dose-response meta-analysis. Nutr. Res. Pract..

[83] Pereira-Santos M., Costa P.R.F., Assis A.M.O., Santos C.A.S.T., Santos D.B. (2015). Obesity and Vitamin D Deficiency: A Systematic Review and Meta-Analysis. Obes. Rev..

[84] Slusher A.L., McAllister M.J., Huang C.J. (2015). A Therapeutic Role for Vitamin D on Obesity-Associated Inflammation and Weight-Loss Intervention. Inflamm. Res..

[85] Jorde R. (2017). RCTs Are the Only Appropriate Way to Demonstrate the Role of Vitamin D in Health. J. Steroid Biochem. Mol. Biol..

[86] Dominguez L.J., Veronese N., Marrone E., Di Palermo C., Iommi C., Ruggirello R., Caffarelli C., Gonnelli S., Barbagallo M. (2024). Vitamin D and Risk of Incident Type 2 Diabetes in Older Adults: An Updated Systematic Review and Meta-Analysis. Nutrients.

[87] Angellotti E., Pittas A.G. (2017). The Role of Vitamin D in the Prevention of Type 2 Diabetes: To D or Not to D?. Endocrinology.

[88] Garbossa S.G., Folli F. (2017). Vitamin D, Sub-Inflammation and Insulin Resistance: A Window on a Potential Role for the Interaction between Bone and Glucose Metabolism. Rev. Endocr. Metab. Disord..

[89] Weller R.B. (2024). Sunlight: Time for a Rethink?. J. Investig. Dermatol..

[90] Mokhtari E., Hajhashemy Z., Saneei P. (2022). Serum vitamin D levels in relation to hypertension and pre-hypertension in adults: A systematic review and dose-response meta-analysis of epidemiologic studies. Front. Nutr..

[91] Valer-Martinez A., Bes-Rastrollo M., Martinez J.A., Martinez-Gonzalez M.A., Sayon-Orea C. (2024). Vitamin D and the Risk of Developing Hypertension in the SUN Project: A Prospective Cohort Study. Nutrients.

[92] Grant W.B., Boucher B.J., Cheng R.Z., Pludowski P., Wimalawansa S.J. (2025). Vitamin D and Cardiovascular Health: A Narrative Review of Risk Reduction Evidence. Nutrients.

[93] Hsu B.-G., Wang Y.-C., Wu D.-A., Chen M.-C. (2024). Serum 25-Hydroxyvitamin D Level Is Positively Associated with Vascular Reactivity Index in Patients with Type 2 Diabetes Mellitus. Nutrients.

[94] Beveridge L.A., Struthers A.D., Khan F., Jorde R., Scragg R., Macdonald H.M., Alvarez J.A., Boxer R.S., Dalbeni A., Gepner A.D. (2015). Effect of vitamin D supplementation on blood pressure: A systematic review and meta-analysis incorporating individual patient data. JAMA Intern. Med..

[95] Di Chio C., Previti S., De Luca F., Allegra A., Zappalà M., Ettari R. (2022). Drug combination studies of PS-1 and quercetin against rhodesain of *Trypanosoma brucei rhodesiense*. Nat. Prod. Res..

[96] Degotte G., Pendeville H., Di Chio C., Ettari R., Pirotte B., Frédérich M., Francotte P. (2023). Dimeric Polyphenols to Pave the Way for New Antimalarial Durgs. RSC Med. Chem..

[97] Santana-Galvez J., Cisneros-Zevallos L., Jacobo-Velazquez D.A. (2017). Chlorogenic acid: Recent advances on its dual role as a food additive and a nutraceutical against metabolic syndrome. Molecules.

[98] Momtazi A.A., Derosa G., Maffioli P., Banach M., Sahebkar A. (2016). Role of microRNAs in the therapeutic effects of curcumin in non-cancer diseases. Mol. Diagn. Ther..

[99] Gordon O.N., Luis P.B., Sintim H.O., Schneider C. (2015). Unraveling curcumin degradation: Autoxidation proceeds through spiroepoxide and vinylether intermediates en route to the main bicyclopentadione. J. Biol. Chem..

[100] Di Chio C., Previti S., De Luca F., Bogacz M., Zimmer C., Wagner A., Schirmeister T., Zappalà M., Ettari R. (2022). Drug Combination Studies of the Dipeptide Nitrile CD24 with Curcumin: A New Strategy to Synergistically Inhibit Rhodesain of *Trypanosoma brucei rhodesiense*. Int. J. Mol. Sci..

[101] Ettari R., Previti S., Di Chio C., Maiorana S., Allegra A., Schirmeister T., Zappalà M. (2020). Drug synergism: Studies of combination of RK-52 and curcumin against rhodesain, cysteine protease of *Trypanosoma brucei rhodesiense*. ACS MedChemLett..

[102] Di Chio C., Previti S., Totaro N., De Luca F., Allegra A., Schirmeister T., Zappalà M., Ettari R. (2023). Dipeptide Nitrile CD34 with Curcumin: A New Improved Combination Strategy to Synergistically Inhibit Rhodesain of *Trypanosoma brucei rhodesiense*. Int. J. Mol. Sci..

[103] Di Chio C., Previti S., Starvaggi J., De Luca F., Calabrò M.L., Zappalà M., Ettari R. (2024). Drug Combination Studies of Isoquinolinone AM12 with Curcumin or Quercetin: A New Combination Strategy to Synergistically Inhibit 20S Proteasome. Int. J. Mol. Sci..

[104] Di Chio C., Starvaggi J., Previti S., De Luca F., Natale B., Cosconati S., Schirmeister T., Zappalà M., Ettari R. (2025). Synthesis and Combination Studies of Novel Dipeptide Nitriles with Curcumin for a Potent Synergistic Action Against Rhodesain, Cysteine Protease of *Trypanosoma brucei rhodesiense*. Pharmaceuticals.

[105] Sahebkar A. (2013). Why it is necessary to translate curcumin into clinical practice for the prevention and treatment of metabolic syndrome?. Biofactors.

[106] Sun D., Wang J., Li X., Peng J., Wang S. (2026). Advances and Perspectives in Curcumin Regulation of Systemic Metabolism: A Focus on Multi-Organ Mechanisms. Antioxidants.

[107] Konaktchieva M., Stojchevski R., Hadzi-Petrushev N., Gagov H., Konakchieva R., Mitrokhin V., Kungulovski G., Mladenov M., Avtanski D. (2025). Curcumin and Tetrahydrocurcumin as Multi-Organ Modulators of the Adipose Tissue–Gut–Liver Axis: Mechanistic Insights, Therapeutic Potential, and Translational Challenges. Pharmaceuticals.

[108] Lamichhane G., Godsey T.J., Liu J., Franks R., Zhang G., Emerson S.R., Kim Y. (2025). Twelve-Week Curcumin Supplementation Improves Glucose Homeostasis and Gut Health in Prediabetic Older Adults: A Pilot, Double-Blind, Placebo-Controlled Trial. Nutrients.

[109] Yang Y.S., Su Y.F., Yang H.W., Lee Y.H., Chou J.I., Ueng K.C. (2014). Lipid-lowering effects of curcumin in patients with metabolic syndrome: A randomized, double-blind, placebo-controlled trial. Phytother. Res..

[110] Panahi Y., Hosseini M.S., Khalili N., Naimi E., Simental-Mendía L.E., Majeed M., Sahebkar A. (2015). Antioxidant and anti-inflammatory effects of curcuminoid–piperine combination in subjects with metabolic syndrome: A randomized controlled trial and an updated meta-analysis. Clin. Nutr..

[111] Lin X.L., Liu M.H., Hu H.J., Feng H.R., Fan X.J., Zou W.W., Pan Y.Q., Peng D.Q. (2015). Curcumin enhanced cholesterol efflux by upregulating ABCA1 expression through AMPK–SIRT1–LXRα signaling in THP-1 macrophage-derived foam cells. DNA Cell Biol..

[112] Cao Z., Yang J., Mai H., Hong X., Xiaobing C., Feng D. (2025). Dietary curcumin prevents hypercholesterolemia by inhibiting the transcriptional activity of SREBP-2 and HNF1α and reducing intestinal and hepatic NPC1L1 expression in high-fat diet-fed hamsters. Nutr. Metab..

[113] Tai M.H., Chen P.K., Chen P.Y., Wu M.J., Ho C.T., Yen J.H., Wang C.J. (2014). Curcumin enhances cell-surface LDLR level and promotes LDL uptake through downregulation of PCSK9 gene expression in HepG2 cells. Mol. Nutr. Food Res..

[114] Momtazi A.A., Banach M., Pirro M., Katsiki N., Sahebkar A. (2017). Regulation of PCSK9 by nutraceuticals. Pharmacol. Res..

[115] Unhapipatpong C., Julanon N., Shantavasinkul P.C., Polruang N., Numthavaj P., Thakkinstian A. (2025). An Umbrella Review of Systematic Reviews and Meta-Analyses of Randomized Controlled Trials Investigating the Effect of Curcumin Supplementation on Lipid Profiles. Nutr. Rev..

[116] Hasanzadeh S., Read M.I., Bland A.R., Majeed M., Jamialahmadi T., Sahebkar A. (2020). Curcumin: An inflammasome silencer. Pharmacol. Res..

[117] Alidadi M., Sahebkar A., Eslami S., Vakilian F., Jarahi L., Alinezhad-Namaghi M., Arabi S.M., Vakili S., Tohidinezhad F., Nikooiyan Y. (2021). The effect of curcumin supplementation on pulse wave velocity in patients with metabolic syndrome: A randomized, double-blind, placebo-controlled trial. Adv. Exp. Med. Biol..

[118] Jäger R., Lowery R.P., Calvanese A.V., Joy J.M., Purpura M., Wilson J.M. (2014). Comparative absorption of curcumin formulations. Nutr. J..

[119] Vashisht M., Rani P., Onteru S.K., Singh D. (2017). Curcumin encapsulated in milk exosomes resists human digestion and possesses enhanced intestinal permeability in vitro. Appl. Biochem. Biotechnol..

[120] Ashtary-Larky D., Rezaei Kelishadi M., Bagheri R., Moosavian S.P., Wong A., Davoodi S.H., Khalili P., Dutheil F., Suzuki K., Asbaghi O. (2021). The Effects of Nano-Curcumin Supplementation on Risk Factors for Cardiovascular Disease: A GRADE-Assessed Systematic Review and Meta-Analysis of Clinical Trials. Antioxidants.

[121] Cai Y., Yang Q., Yu Y., Yang F., Bai R., Fan X. (2023). Efficacy and Underlying Mechanisms of Berberine against Lipid Metabolic Diseases: A Review. Front. Pharmacol..

[122] Sun A., Yang H., Li T., Luo J., Zhou L., Chen R., Han L., Lin Y. (2024). Molecular Mechanisms, Targets and Clinical Potential of Berberine in Regulating Metabolism: A Review Focussing on Databases and Molecular Docking Studies. Front. Pharmacol..

[123] Caliceti C., Rizzo P., Ferrari R., Fortini F., Aquila G., Leoncini E., Taddei M., Cicero A.F.G. (2017). Novel role of the nutraceutical bioactive compound berberine in lectin-like OxLDL receptor 1-mediated endothelial dysfunction in comparison to lovastatin. Nutr. Metab. Cardiovasc. Dis..

[124] Zieniuk B., Pawełkowicz M. (2025). Berberine as a Bioactive Alkaloid: Multi-Omics Perspectives on Its Role in Obesity Management. Metabolites.

[125] Ferri N., Corsini A., Sirtori C.R., Ruscica M. (2017). PPAR-α agonists are still on the rise: An update on clinical and experimental findings. Expert Opin. Investig. Drugs.

[126] Ji L., Ma J., Ma Y., Zhang X., Liu Y., Li W., Sun Q., Chen R., Huang H., Wang X. (2025). Berberine Ursodeoxycholate for the Treatment of Type 2 Diabetes: A Randomized Clinical Trial. JAMA Netw. Open.

[127] Mbara K.C., Kheoane P.S., Tarirai C. (2025). Targeting AMPK Signaling: The Therapeutic Potential of Berberine in Diabetes and Its Complications. Pharmacol. Res. Mod. Chin. Med..

[128] Yang G., Li X., Li X., Li Y., Li W., Zhou Z. (2012). Traditional Chinese medicine in cancer care: A review of case series published in the Chinese literature. Evid. Based Complement. Alternat. Med..

[129] Zhang H., Xiong P., Zheng T., Hu Y., Guo P., Shen T., Zhou X. (2025). Combination of Berberine and Evodiamine Alleviates Obesity by Promoting Browning in 3T3-L1 Cells and High-Fat Diet-Induced Mice. Int. J. Mol. Sci..

[130] Baek Y.S., Kim S., Lee E., Lee S. (2025). Allosteric Inhibition of the PCSK9–LDLR Interaction: Structural Insights for Small-Molecule Design. Phys. Chem. Chem. Phys..

[131] Ferri N., Corsini A., Macchi C., Ruscica M., Sirtori C.R., Banach M., Pirro M. (2016). Proprotein convertase subtilisin kexin type 9 and high-density lipoprotein metabolism: Experimental animal models and clinical evidence. Transl. Res..

[132] Huh J., Kim H. (2025). Naturally Occurring PCSK9 Inhibitors: An Updated Review. Molecules.

[133] Dong B., Li H., Singh A.B., Dong H., Liu J., DeSmet M., Zhang L., Mahata S.K., Rao R. (2015). Inhibition of PCSK9 transcription by berberine involves down-regulation of hepatic HNF1α protein expression through the ubiquitin–proteasome degradation pathway. J. Biol. Chem..

[134] Ruscica M., Ricci C., Macchi C., Magni P., Ferri N., Corsini A., Banach M., Pirro M. (2016). Suppressor of cytokine signaling-3 (SOCS-3) induces Proprotein Convertase Subtilisin Kexin Type 9 (PCSK9) expression in hepatic HepG2 cell line. J. Biol. Chem..

[135] Caliceti C., Franco P., Spinozzi S., Rizzo P., Ruscica M., Corsini A., Ferri N. (2016). Berberine: New insights from pharmacological aspects to clinical evidence in the management of metabolic disorders. Curr. Med. Chem..

[136] Asghari P., Babaei A., Zamanian N., Eshtivani E.N. (2025). Berberine’s impact on health: Comprehensive biological, pharmacological, and nutritional perspectives. Metabol. Open.

[137] Faulkner R.A., Yang Y., Tsien J., Qin T., DeBose-Boyd R.A. (2024). Direct binding to sterols accelerates endoplasmic reticulum-associated degradation of HMG CoA reductase. Proc. Natl. Acad. Sci. USA.

[138] Childress L., Gay A., Zargar A., Fahrenkrug S.C., Durrant A., Baker D. (2013). Review of red yeast rice content and current Food and Drug Administration oversight. J. Clin. Lipidol..

[139] EFSA Panel on Dietetic Products, Nutrition, and Allergies (NDA) (2011). Scientific opinion on the substantiation of health claims related to monacolin K from red yeast rice and maintenance of normal blood LDL cholesterol concentrations (ID 1648, 1700) pursuant to Article 13(1) of Regulation (EC) No. 1924/2006. EFSA J..

[140] Yang C., Wu Y., Qian J., Li J.J. (2024). A systematic, updated review of Xuezhikang, a domestically developed lipid-lowering drug, in the application of cardiovascular diseases. Acta Pharm. Sin. B.

[141] Cicero A.F.G., Morbini M., Rosticci M., Grandi E., Borghi C. (2016). Middle-term dietary supplementation with red yeast rice plus coenzyme Q10 improves lipid pattern, endothelial reactivity and arterial stiffness in moderately hypercholesterolemic subjects. Ann. Nutr. Metab..

[142] Fogacci F., Giovannini M., Di Micoli V., Grandi E., Veronesi M., Borghi C., Cicero A.F.G. (2023). Evaluation of the effect of a dietary supplementation with a red yeast rice and fish oil-containing nutraceutical on lipid pattern, high-sensitivity C-reactive protein, and endothelial function in moderately hypercholesterolaemic subjects: A double-blind, placebo-controlled, randomized clinical trial. Arch. Med. Sci. Atheroscler. Dis..

[143] Zhu X.Y., Li P., Yang Y.B., Wang D.D., Yang Z., Wang S., Zhang L. (2013). Xuezhikang, extract of red yeast rice, improved abnormal hemorheology, suppressed caveolin-1 and increased eNOS expression in atherosclerotic rats. PLoS ONE.

[144] Verhoeven V., Van der Auwera A., Van Gaal L., D’Hooge J., Desmet S., Vanhorebeek I., Mathieu C., Van Gaal L. (2015). Can red yeast rice and olive extract improve lipid profile and cardiovascular risk in metabolic syndrome?: A double blind, placebo controlled randomized trial. BMC Complement. Altern. Med..

[145] Pirro M., Mannarino M.R., Bianconi V., Savarese G., Bagaglia F., Marchesi S., Baratta F., Mannarino E., Siepi D., Simental-Mendía L.E. (2016). The effects of a nutraceutical combination on plasma lipids and glucose: A systematic review and meta-analysis of randomized controlled trials. Pharmacol. Res..

[146] Arnaboldi F., Busnelli M., Cornaghi L., Gori L., Conti S., Ronda N., Caldi E., Sirtori C.R., Corsini A., Macchi C. (2015). High-density lipoprotein deficiency in genetically modified mice deeply affects skin morphology: A structural and ultrastructural study. Exp. Cell Res..

[147] Ruscica M., Gomaraschi M., Mombelli G., Calabresi L., Ferri N., Corsini A. (2014). Nutraceutical approach to moderate cardiometabolic risk: Results of a randomized, double-blind and crossover study with Armolipid Plus. J. Clin. Lipidol..

[148] Spigoni V., Aldigeri R., Antonini M., Micheli M.M., Fantuzzi F., Fratter A., Pellizzato M., Derlindati E., Zavaroni I., Bonadonna R.C. (2017). Effects of a New Nutraceutical Formulation (Berberine, Red Yeast Rice and Chitosan) on Non-HDL Cholesterol Levels in Individuals with Dyslipidemia: Results from a Randomized, Double Blind, Placebo-Controlled Study. Int. J. Mol. Sci..

[149] Trogkanis E., Karalexi M.A., Sergentanis T.N., Kornarou E., Vassilakou T. (2024). Safety and Efficacy of the Consumption of the Nutraceutical “Red Yeast Rice Extract” for the Reduction of Hypercholesterolemia in Humans: A Systematic Review and Meta-Analysis. Nutrients.

[150] Musumeci O., Drago S.F.A. (2025). Update on statin-associated myopathy symptoms in the view of new clinical management strategies. Curr. Opin. Neurol..

[151] Čakir M.C., Özden S. (2025). The Role of Global DNA Methylation in Citrinin-Induced Toxicity: In Vitro and In Silico Approach. Toxicol. Rep..

[152] Pascual-Ahuir A., Vanacloig-Pedros E., Proft M. (2014). Toxicity mechanisms of the food contaminant citrinin: Application of a quantitative yeast model. Nutrients.

[153] Kumarasinghe H.S., Gunathilaka M.D.T.L. (2024). A systematic review of fucoxanthin as a promising bioactive compound in drug development. Phytochem. Lett..

[154] Millan J., Cicero A.F.G., Torres F., Anguera A. (2016). Effects of a nutraceutical combination containing berberine (BRB), policosanol, and red yeast rice (RYR), on lipid profile in hypercholesterolemic patients: A meta-analysis of randomized controlled trials. Clin. Investig. Arterioscler..

[155] ElSayed N.A., Aleppo G., Aroda V.R., Bannuru R.R., Brown F.M., Bruemmer D., Collins B.S., Hilliard M.E., Isaacs D., Johnson E.L. (2023). Classification and diagnosis of diabetes: Standards of care in diabetes—2023. Diabetes Care.

[156] Figueira N., Curtain F., Beck E., Grafenauer S. (2019). Consumer Understanding and Culinary Use of Legumes in Australia. Nutrients.

[157] Kamle M., Mahato D.K., Gupta A., Pandhi S., Sharma N., Sharma B., Mishra S., Arora S., Selvakumar R., Saurabh V. (2022). Citrinin Mycotoxin Contamination in Food and Feed: Impact on Agriculture, Human Health, and Detection and Management Strategies. Toxins.

[158] Younes M., Aggett P., Aguilar F., Crebelli R., Dusemund B., Filipič M., Frutos M.J., Galtier P., Gott D., EFSA Panel on Food Additives and Nutrient Sources added to Food (ANS) (2018). Scientific opinion on the safety of monacolins in red yeast rice. EFSA J..

[159] Siddiqui R.A., Moghadasian M.H. (2020). Nutraceuticals and Nutrition Supplements: Challenges and Opportunities. Nutrients.

[160] Singh C., Pandey A., Gupta P., Sharma S., Agrawal P., Rawat A.K.S., Singh D. (2020). Nanotechnology-based strategies to improve the bioavailability and therapeutic efficacy of polyphenols. Nutrients.

